# Hookworm infection modulates lung and intestinal transcriptomic responses to SARS-CoV-2 in Syrian hamsters

**DOI:** 10.3389/fimmu.2025.1701728

**Published:** 2025-11-05

**Authors:** Bruce A. Rosa, Mahdiyeh Bigham, Tamarand L. Darling, Ashutosh Arun, Kumar Sachin Singh, John Martin, Adrianus C. M. Boon, Makedonka Mitreva

**Affiliations:** ^1^ Division of Infectious Diseases, Department of Medicine, Washington University School of Medicine, St. Louis, MO, United States; ^2^ Department of Molecular Microbiology, Washington University School of Medicine, St. Louis, MO, United States; ^3^ Department of Pathology and Immunology, Washington University School of Medicine, St. Louis, MO, United States; ^4^ Department of Genetics, Washington University School of Medicine, St. Louis, MO, United States; ^5^ McDonnell Genome Institute, Washington University School of Medicine, St. Louis, MO, United States

**Keywords:** hookworm, SARS-CoV-2, coinfection, host response, transcriptomics, helminth-virus interaction, immune modulation

## Abstract

**Background:**

Helminth infections are widespread in resource-limited settings, and modulate host immune responses, with potential implications for viral coinfections. Intestinal helminths can alter susceptibility to respiratory viruses, but the mechanisms influencing SARS-CoV-2 infection outcomes remain poorly understood.

**Methods:**

Using the Syrian hamster model, we investigated the impact of prior infection with the human hookworm *Ancylostoma ceylanicum* on host responses to SARS-CoV-2. Tissue-specific transcriptional responses were compared among four groups: naive, hookworm-only, SARS-CoV-2-only, and coinfected with both pathogens, 3 and 6 days post-viral infection. Viral titers and weight loss were assessed, and RNA-seq transcriptome profiles from lung and intestinal tissues were interrogated to identify differentially expressed genes and cellular pathways.

**Results:**

Prior hookworm infection did not significantly alter viral titers or weight loss compared to SARS-CoV-2 infection alone, but distinct transcriptional signatures compared were identified compared to either single infection. Coinfection uniquely differentially regulated hematopoiesis and B cell-associated genes (e.g., *ATF5*, *IGHM*, *JCHAIN*) in the lungs, and immune and stress response pathways and inflammation-associated genes (e.g. *FOLR2*, *PLA2GF*, *FABP3*) in the intestine. Genes and pathways differentially regulated by SARS-CoV-2 alone, but with attenuated transcriptional responses in the lungs of coinfected hamsters were observed, including the loss of upregulation of toll-like receptor signaling and previously proposed host biomarkers for COVID-19 severity (*CHI3L1*, *HMOX1*), Long COVID (*FCG4*/*FCGR3A* and *FST*) and mortality (*FST*). In the intestine, hookworm-associated suppression of type I interferon-related genes (*TAP1*, *IRF7*) was reversed with SARS-CoV-2 coinfection, highlighting pathogen-specific modulation of innate antiviral signaling. Genes and pathways consistently differentially regulated by with SARS-CoV-2 were consistent with expectations, and many hemoglobin pathways were differentially regulated with hookworm in the intestine. CIBERSORT analysis was estimated relative leukocyte abundances in each sample cohort.

**Conclusion:**

Our findings demonstrate that *A. ceylanicum* infection reshapes host transcriptional responses to SARS-CoV-2 in a tissue-specific manner, enhancing B cell immunity in the lung while driving intestinal inflammation. Hookworm-induced immune modulation attenuated key SARS-CoV-2-responsive genes and pathways, suggesting potential mechanisms for reduced disease severity observed in helminth-endemic regions. These findings establish a molecular framework to better understand helminth, SARS-CoV-2 and host immune interactions, with relevance for other respiratory viral infections.

## 1 Introduction

The SARS-CoV-2 pandemic presented a significant global health challenge, resulting in 777 million total reported infection cases and more than 7 million deaths reported worldwide (1, 2). Individuals aged 65 and older remain at risk for infection, as they are severely affected by severe COVID-19, accounting for 80% of hospitalizations and exhibiting a 23-fold increase in mortality compared to younger age groups. Age-associated alterations likely influence this increased susceptibility in immune system efficiency, genetic regulation, cellular metabolism, and inflammatory processes (3). Contrary to early predictions of severe outcomes in Africa, the continent has recorded comparatively lower infection and mortality rates from the start of the pandemic in 2020 to 2022-2023. While the median age in Africa is lower compared to other continents, coinfections with other pathogens may have reduced the impact of the COVID-19 pandemic. Emerging evidence suggests that coinfection with malaria can alter disease outcomes, potentially improving survival rates in some cases (4). This interaction highlights the complex immune modulation induced by chronic malaria parasite exposure, which may influence the progression of viral infections. Understanding these coinfection dynamics could provide new insights for disease management and therapeutic strategies in regions burdened by both malaria and viral epidemics. Specifically, hookworm infections affect approximately 472 million people in resource-limited rural areas, resulting in over 4 million disability-adjusted life years (DALYs) lost annually and imposing an economic burden exceeding US$100 billion per year (5).

Chronic and acute hookworm infections can modulate the host immune system by inducing a type 2 (Th2) immune response and overall immune functionality (6). Helminths modify the immune system by secretion of immunomodulatory molecules that regulate host innate and adaptive immune responses (7, 8). These changes can significantly impact disease progression and clinical presentation during coinfection with other pathogens including respiratory viruses. Before 2019, helminth infections were demonstrated to confer protection against the influenza and respiratory syncytial virus (RSV) in mouse disease models. This protective effect was linked to the activation of effector CD4^+^ and CD8^+^ T cells (9), which enhance immune responses by upregulating type I interferons, pro-inflammatory cytokines, and antiviral factors such as interferon stimulated genes (*ISG*), and *CRAMP* gene expression in the lungs and duodenum (10). It was shown that coinfection of mice with the helminths *Trichinella* sp*iralis* or *Heligmosomoides polygyrus* and influenza virus alters immune responses and lung pathology (11).

In a study of 751 SARS-CoV-2 patients in Africa, infection with intestinal helminths was associated with a significantly lower probability of developing severe COVID-19 (12), and chronic helminth infections are thought to suppress SARS-CoV-2 entry and hyperinflammation, by attenuating inflammatory signaling pathways and suppressing the release of pro-inflammatory cytokines (13). Additionally, helminth extracts applied *in vitro* to PBMCs isolated from SARS-CoV-2 patients modulate immune reactivity to SARS-CoV-2 peptides by suppressing reactive CD4+ helper T cells but maintaining reactive CD8+ cytotoxic T cells (14).

Animal models to study helminth and SARS-CoV-2 coinfection present an opportunity to better understand these mechanisms using different helminth species. Previous research in hamsters has investigated specific aspects of COVID-19 coinfection with parasitic helminths, such as *Schistosoma* (15), and assessed the impacts of the COVID-19 pandemic on neglected tropical diseases (NTDs) (16). In mice, prior infection with the lung-migrating rodent helminth *Nippostrongylus brasiliensis* enhances the immune system’s response to SARS-CoV-2, improving viral clearance and reducing disease severity by boosting CD8+ T cell recruitment and activation, a process that depends on macrophages in mice (17). In another mouse study, infection with the pork tapeworm *T.* sp*iralis* indicated that anti-inflammatory markers from its secreted products may mitigate adverse pathology from SARS-CoV-2 infection (18). While informative, mice are not natural hosts for these viruses or helminths, and laboratory mice are pathogen-free, which has a significant impact on disease induced by viruses (19, 20) and potentially worms (11, 19, 21).

In this study, we aimed to investigate the impact of experimental infection with the human-parasitic intestinal hookworm *Ancylostoma ceylanicum* using a Syrian hamster SARS-CoV-2 model. This experimental model represents the first helminth-SARS-CoV-2 study to examine whole-transcriptome profiling of host responses, facilitating the discovery of novel genes and pathways involved beyond targeted immune profiling performed in previous studies. We utilized separate single-infection and coinfection cohorts to analyze the transcriptome responses of both the lung and the intestine (at 3 days and 6 days post-SARS-CoV-2 infection), to comprehensively characterize the distinct host responses associated uniquely with coinfection in a tissue-specific manner. The results identify known and published differentially expressed genes, as well as many novel responsive genes and pathways of interest for future studies.

## 2 Materials and methods

### 2.1 Ethics statements

All animal experiments were carried out under protocols approved by Washington University School of Medicine Institutional Animal Care and Use Committees (assurance number A3381-01). All housing and care of laboratory animals conformed to the National Institutes of Health (NIH) Guide for the Care and Use of Laboratory Animals in Research (see 18-F22) and all requirements and regulations issued by the United States Department of Agriculture (USDA), including regulations implementing the Animal Welfare Act (P.L. 89-544) as amended (see 18-F23).

### 2.2 Cells and viruses

Vero cells expressing human angiotensin converting enzyme 2 (ACE2) and transmembrane protease serine 2 (TMPRSS2) (Vero-hACE2-hTMPRSS2, gift of Adrian Creanga and Barney Graham, NIH) were cultured at 37 °C in Dulbecco’s Modified Eagle medium (DMEM) supplemented with 10% fetal bovine serum (FBS), 10 mM HEPES (pH 7.3), 2 mM L-glutamine, 100 U/mL Penicillin, 100 µg/mL Streptomycin, and 10 µg/mL of puromycin. Vero E6 cells expressing human TMPRSS2 (Vero-hTRMPRSS2) were cultured at 37 °C in DMEM supplemented with 10% FBS, 10 mM HEPES (pH 7.3), 2 mM L-glutamine, 100 U/mL Penicillin, 100 µg/mL Streptomycin, and 5 µg/mL of blasticidin. Vero-hACE2-hTRMPSS2 are used to titrate stocks and tissues and Vero-hTRMPSS2 cells are used to generate virus stocks. SARS-CoV-2 (strain 2019-nCoV/USA-WA1/2020) was obtained from the US Centers for Disease Control (CDC) and the virus stock sequence was confirmed by next-generation sequencing prior to *in vitro* or *in vivo* use. All work with infectious SARS-CoV-2 was performed in Institutional Biosafety Committee approved BSL-3 and ABSL3 facilities at Washington University School of Medicine using appropriate positive pressure air respirators and protective equipment.

### 2.3 Hamster infection and euthanasia

**Figure 1:**
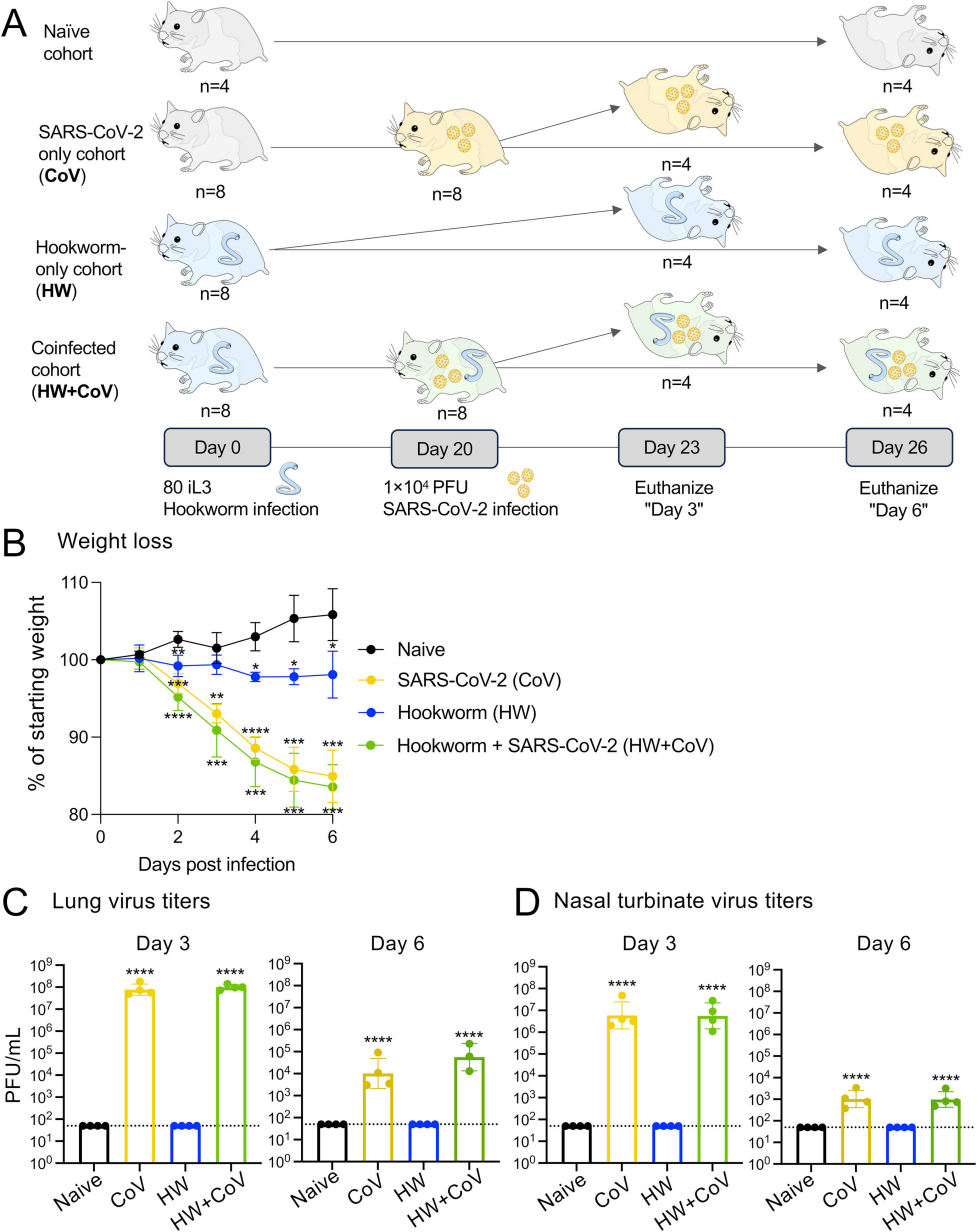
Overview of experimental cohorts, and the effects of infection on hamster weights and virus titers. **(A)** A total of 28 Syrian hamsters were allocated into four distinct cohorts to represent different infection conditions: Naive, CoV, HW and HW+CoV. Hamsters in the HW and HW+CoV cohorts were initially infected with 80 third-stage larvae (iL3). After a period of 20 days, hamsters in the CoV and HW+CoV cohorts were subsequently infected with 1.0x10^4^ plaque-forming units (PFU) of SARS-CoV-2 (strain 2019-nCoV/USA-WA1/2020). Tissue samples from the small intestine and lung were collected at two time points: 3 days (day 23) and 6 days (day 26) after the CoV infection timepoint (referred to as day 3 and day 6 for the analysis). **(B)** Hamster body weight for each of the three infection cohorts (CoV, HW and HW+CoV) was compared to the naive cohort at each day of infection using a two-way ANOVA mixed-effects model with Dunnett’s multiple comparison testing. After hamsters were sacrificed (at day 3 and day 6), SARS-CoV-2 virus titers were measured in **(C)** lung tissue and **(D)** nasal turbinates, compared to the naive cohort using a one-way ANOVA with Dunnett’s multiple comparison test. Error bars represent standard deviation values; *P ≤ 0.05, **P ≤ 0.01, ***P ≤ 10^-3^, ****P ≤ 10^-4^.

Twenty-eight male hamsters, aged five to six weeks old, were purchased from Charles River Laboratory and split into four cohorts to represent uninfected (naive), hookworm-only (HW), virus-only (CoV), and worm-virus coinfected (HW+CoV) cohorts. The HW and HW+CoV cohorts were inoculated by oral gavage with 80 infected L3 (iL3) at day 0. Twenty days post hookworm infection, both the CoV and HW+CoV cohorts were intranasally inoculated with 1.0×10^4^ PFU SARS-CoV-2 (strain 2019-nCoV/USA-WA1/2020) in 100 µL following isoflurane sedation. Weights were recorded daily for all cohorts after viral inoculation. At day 23 and 26, hamsters were euthanized using appropriate technique, equipment, and agents. Compressed CO_2_ gas in cylinders were used as a source of carbon dioxide, allowing for the controlled inflow of gas to the induction chamber. Hamsters to be euthanized were placed in a clean transparent chamber and 100% CO_2_ was introduced at a fill rate of 70% of the chamber volume per minute, to achieve a balanced gas mixture with the existing air in the chamber to fulfill the objective of rapid unconsciousness with minimal distress to the animals. Expected time to unconsciousness was usually within 3–5 minutes. Each hamster was monitored for lack of respiration and faded eye color. CO_2_ was maintained for a minimum of 1 minute after observing these signs to avoid unintended recovery upon exposure to natural CO_2_ concentrations. Upon completion of the procedure, death was confirmed by ascertaining cardiac and respiratory arrest and noting an animal’s fixed and dilated pupils. Organs were dissected as described below and collected for infectious titer and RNA-seq analysis. All sample cohorts, including the control, include four biological replicates. A detailed sample collection timeline is provided in **Figure 1A**.

### 2.4 Infectious viral titer

At time of collection, the right lung lobe and nasal turbinate were collected. Nasal turbinates were collected by removing the skin along the nose and cheeks then cutting the jaw to expose the upper palate. A sagittal incision through the palate exposed the nasal turbinates, which were then removed using blunt forceps. The right lung and nasal turbinates were homogenized in 1.0 mL DMEM and clarified by centrifugation at 1,000×g for five minutes. Plaque assays were performed on Vero-hACE2-hTRMPSS2 cells in 24-well plates. Lung tissue homogenates and nasal turbinate homogenates were serially diluted in cell infection medium (DMEM supplemented with 2% FBS, 10 mM HEPES [pH 7.3] and 2 mM L-glutamine). Two hundred microliters of the diluted virus were added to a single well per dilution per sample. After one hour at 37 °C, the inoculum was aspirated, the cells were washed with PBS, and a 1% methylcellulose overlay in MEM supplemented with 2% FBS was added. Seventy-two hours after virus inoculation, the cells were fixed with 10% formalin, and the monolayer was stained with crystal violet (0.5% w/v in 25% methanol in water) for one hour at 20 °C. The number of plaques were counted and used to calculate the plaque forming units/mL (PFU/mL). Infectious virus titer detected in any of the contact hamster organs was considered a positive transmission event.

### 2.5 RNA preparation and sequencing

At time of collection, the remaining lung lobes and a dissection of the small intestine were collected for RNA-seq analysis. A 4 cm piece of the anterior end of the small intestine (near the duodenum, 1-1.5 cm after the stomach) was cut and opened longitudinally. The contents were washed out with PBS three times, and the piece was split into two 2 cm halves. One half of the small intestine and the right lung was homogenized in 1.0 mL Trizol (Life Technologies) and centrifuged at 1,000×g for 5 minutes. RNA was extracted per manufacturer; briefly, 200 µL chloroform was added to the samples and vortexed. After a short incubation, the samples were centrifuged for 15 minutes at 12,000×g at 4 °C. The aqueous phase was transferred to a new Eppendorf tube and 500 µL isopropanol was added. After a 10-minute incubation, the samples were centrifuged at 4 °C at 12,000×g for 10 minutes. The pellet was resuspended in 75% ethanol and vortexed before centrifugation at 4 °C at 7,500×g for 5 minutes. The pellet was then resuspended in 30 µL RNase-free water and incubated at 55 °C for 15 minutes. RNA was then stored at -80 °C until use. Sequencing was performed by the Genome Technology Access Center at McDonnell Genome Institute (GTAC@MGI) at Washington University in St. Louis School of Medicine, USA. RNA sequencing libraries were generated using Clontech SMART-Seq v4 Ultra Low Input RNA Kit for sequencing and Illumina Nextera XT DNA Library preparation kit following the manufacturer’s protocol. The cDNA libraries were validated using KAPA Biosystems primer premix kit with Illumina-compatible DNA primers and quality was examined using Agilent Tapestation 2200. cDNA libraries were prepared from RNA samples using PolyA selection, and processed cDNA was sequenced on the Illumina NovaSeq S4 platform (paired-end 150bp reads), generating an average of 36.3 million read pairs per sample (see **Supplementary Table S1** **).**

### 2.6 RNA-seq differential expression analysis

**Figure 2:**
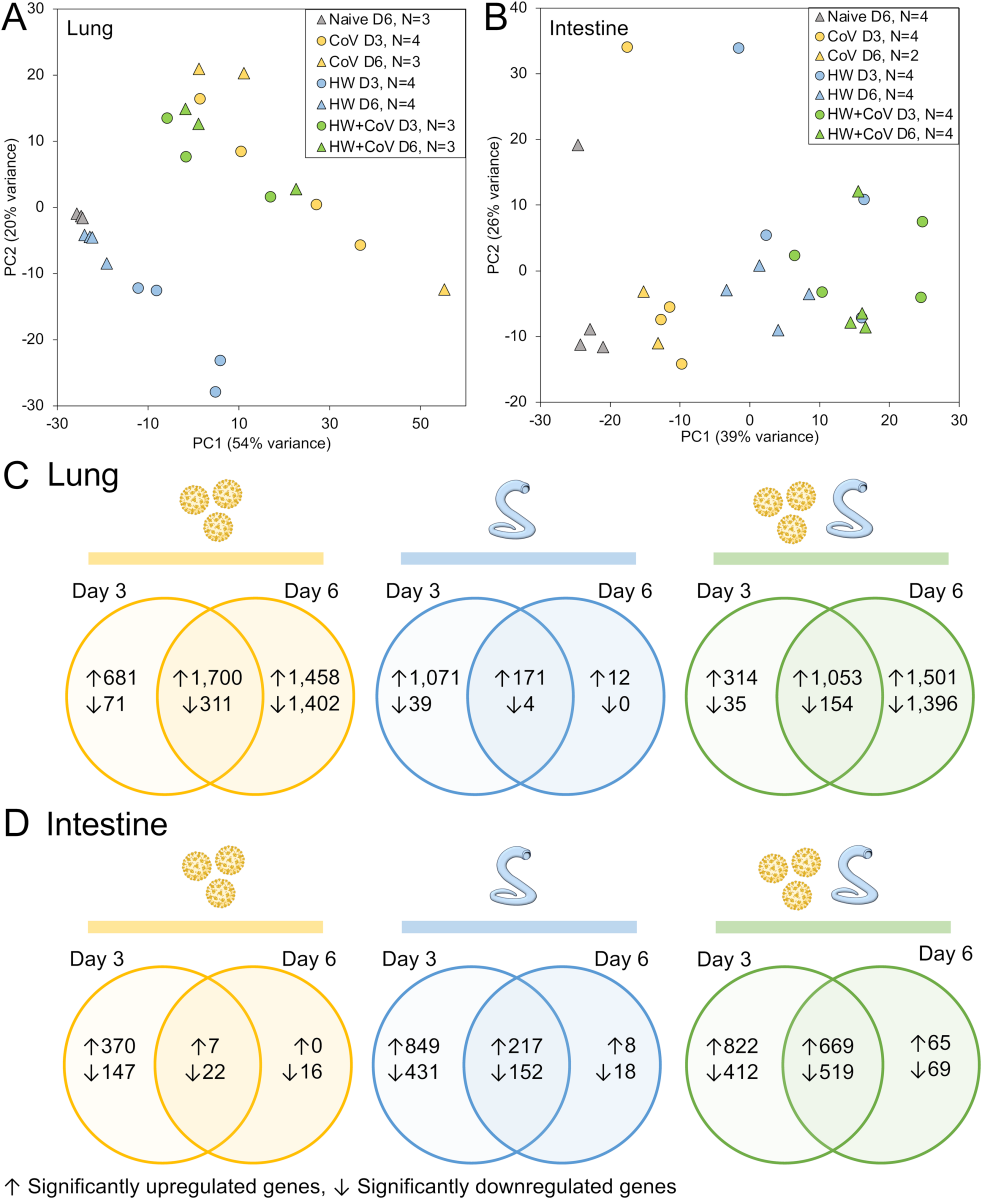
RNA-seq analysis overview. Principal components analysis (PCA) depicting **(A)** lung and **(B)** intestine RNA-seq sample similarity based on overall transcriptome profiles, according to DESeq2 analysis, based on sample groups as described in **Figure 1A**. Sample counts are shown after quality control filtering of RNA-seq sample sets. According to the DESeq2, significantly differentially expressed genes (DEGs) are counted and intersected at the two timepoints for each sample cohort (CoV=yellow, HW=blue, HW+COV=green) in **(C)** the lungs and **(D)** the intestines of the hamsters following infection, compared to the Naive cohort.

**Figure 3:**
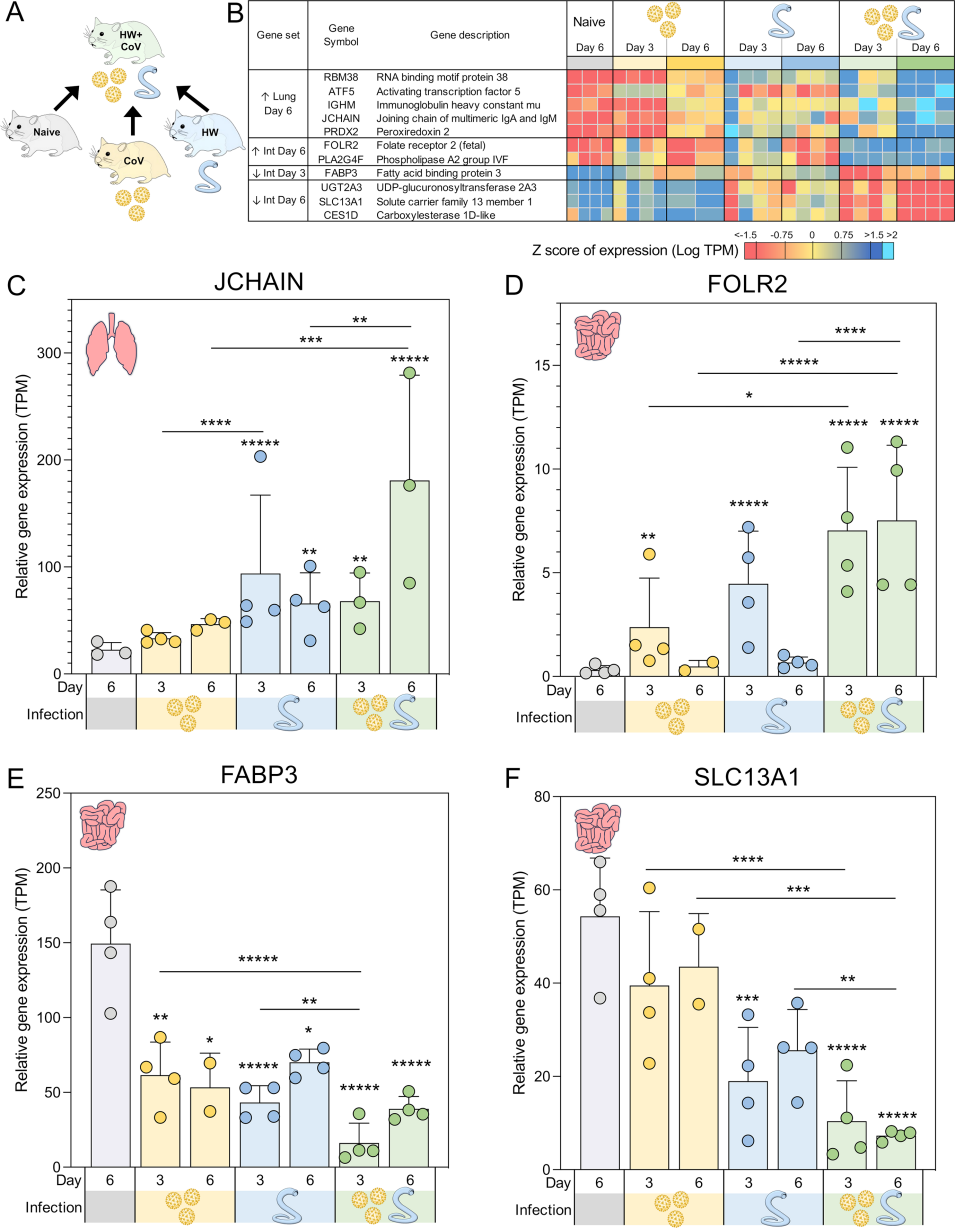
Genes significantly differentially expressed during coinfection vs all other sample groups. **(A)** Schematic of the cohorts being compared, with the HW+CoV coinfection cohort being compared to the other three cohorts, for every tissue and timepoint. **(B)** Heatmap representation of the expression levels of the DEGs of the coinfection group vs all other sample groups in the lungs and intestine (Int). Genes are sorted by FDR-adjusted DESeq2 *P* values. Swarm plot representations of differential expression are shown for **(C)** JCHAIN, **(D)** FOLR2, **(E)** FABP3 and **(F)** SLC13A1, which are genes of interest from panel B, with DESeq2 FDR-corrected *P* values for significance indicated (* *P* ≤ 0.05, ** *P* ≤ 0.01, ***P ≤ 10^-3^, ****P ≤ 10^-4,^*****P ≤ 10^-5^). Asterisks directly above a series indicate significance vs. the uninfected control cohort (grey). Comparisons between infected cohorts were tested on the same days only (i.e., days 3 and 6 are not compared to each other).

**Figure 4:**
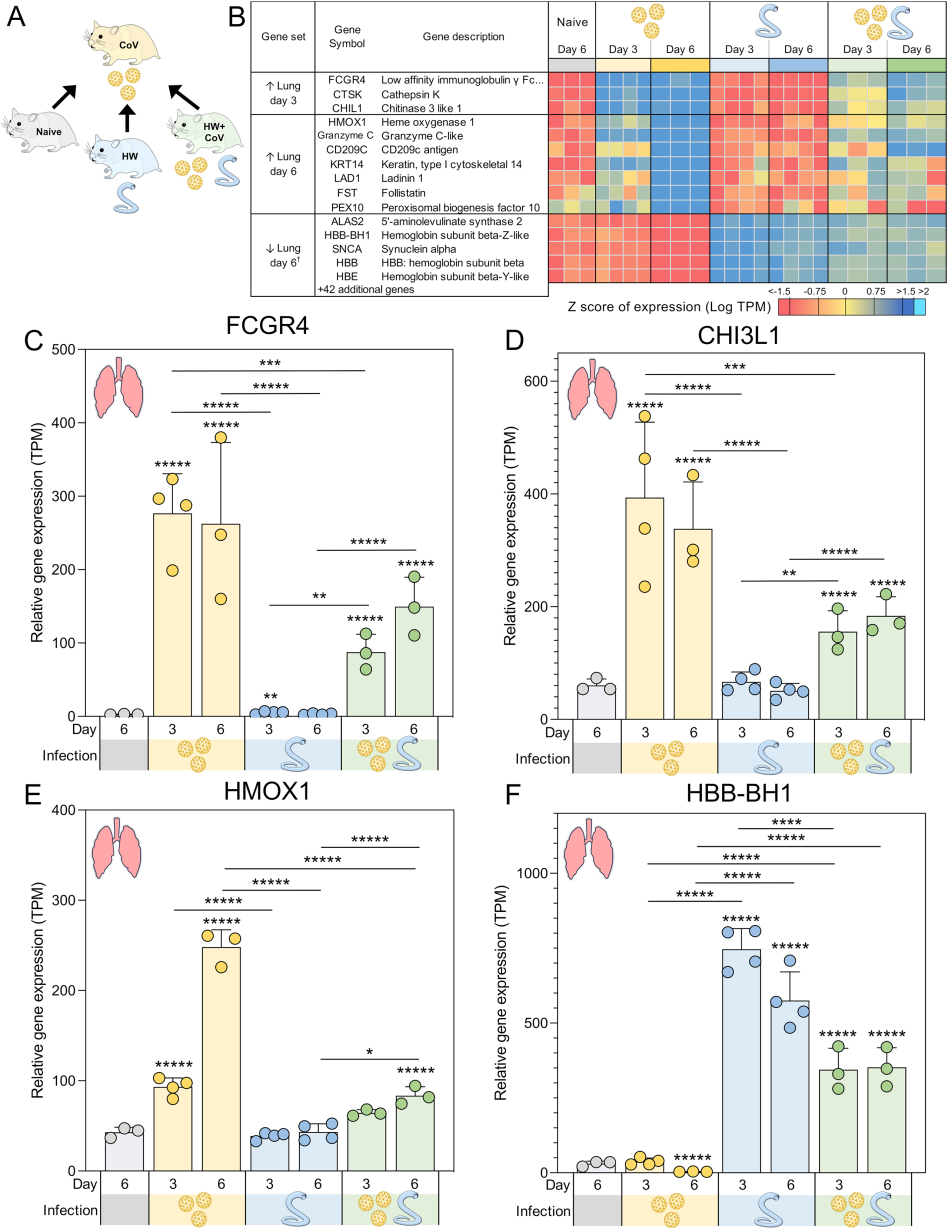
Genes significantly differentially expressed during SARS-CoV-2-only infection vs all other sample groups. **(A)** Schematic of the cohorts being compared, with the CoV single infection cohort being compared to the other three cohorts for every tissue and time point. **(B)** Heatmap representation of the expression levels of gene sets significantly upregulated or downregulated between the SARS-CoV-2-only group and all other sample groups in the lungs. Genes are sorted by DESeq2 FDR-adjusted *P* values. Swarm plot representations of differential expression are shown for **(C)** FCGR4, **(D)** CHI3L1, **(E)** HMOX1 and **(F)** HBB-BH1, which are genes of interest from panel B, with DESeq2 FDR-corrected *P* values for significance indicated (*P ≤ 0.05, **P ≤ 0.01, ***P ≤ 10^-3^, ****P ≤ 10^-4^, *****P ≤ 10^-5^). Asterisks directly above a series indicate significance vs. the uninfected control cohort (grey). Comparisons between infected cohorts were tested on the same days only (i.e., days 3 and 6 are not compared). ^†^Top five most significant of 47 total genes shown.

**Figure 5:**
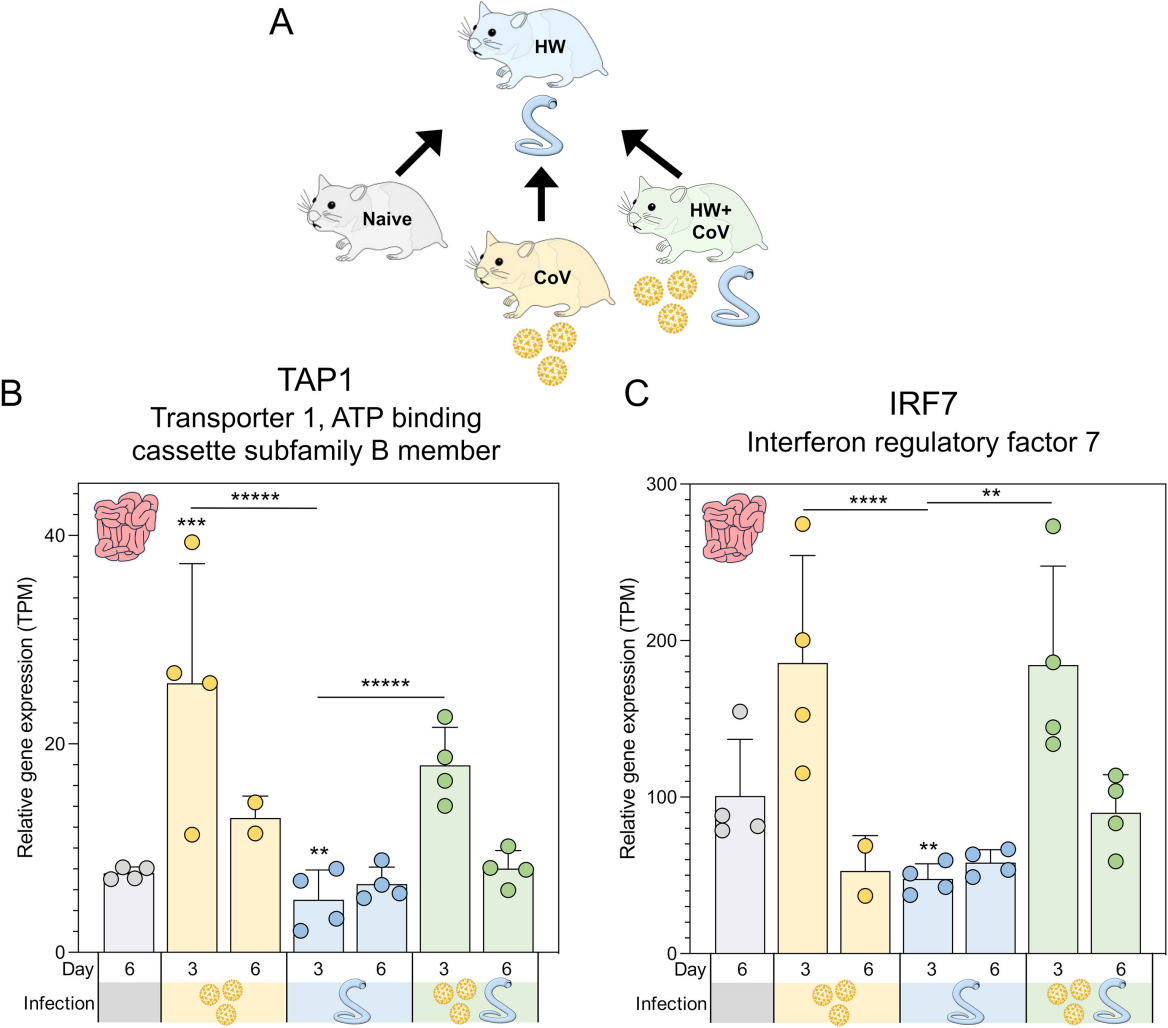
Genes significantly differentially expressed during hookworm-only infection vs all other sample groups. **(A)** Schematic of the cohorts being compared, with the HW single infection cohort being compared to the other three cohorts, for every tissue and timepoint. Swarm plot representations of differential expression are shown for the two genes of interest: **(B)** TAP1 and **(C)** IRF7. *P* values for significance indicated (**P ≤ 0.01, ***P ≤ 10^-3^, ****P ≤ 10^-4^, *****P ≤ 10^-5^). Asterisks directly above a series indicate significance vs. the uninfected control cohort (grey). Comparisons between infected cohorts were tested on the same days only (i.e., days 3 and 6 are not compared to each other).

**Figure 6:**
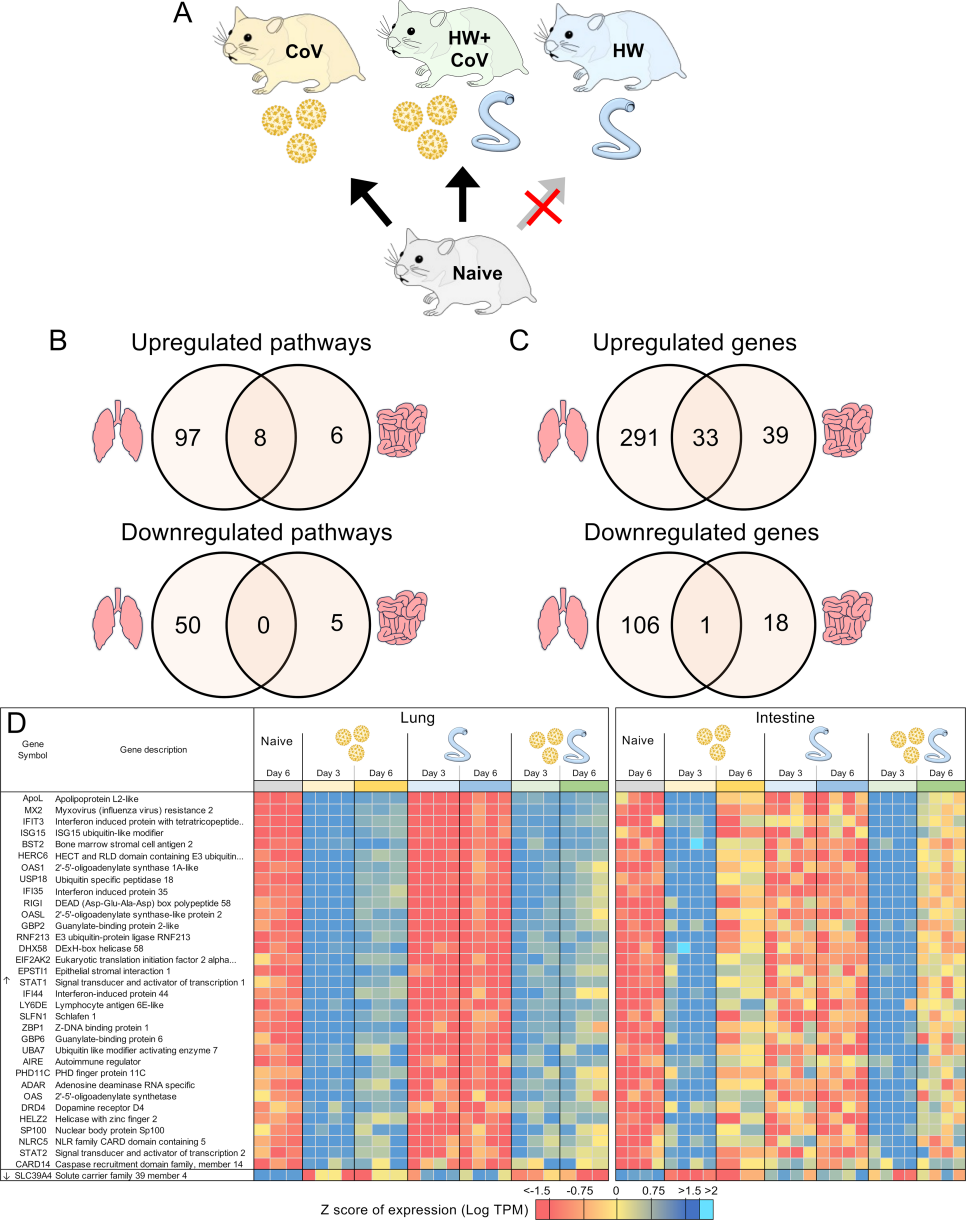
DEGs differentially regulated by SARS-CoV-2 but not by hookworm, regardless of coinfection status. **(A)** Schematic of the cohorts being compared: The comparison indicated by the red X must not be significant for genes to be considered. **(B)** Intersections of pathways of interest enriched among sets of DEGs in both the lung (at both day 3 and day 6) and the intestine (at day 3). **(C)** Intersections of DEGs of interest in both the lung (day 3 and day 6) and the intestine (day 3). **(D)** Heatmap representation of the expression levels of gene sets from the intersections of the Venn diagrams from panel **(C)** Genes are sorted by DESeq2 FDR-adjusted *P* values.

**Figure 7:**
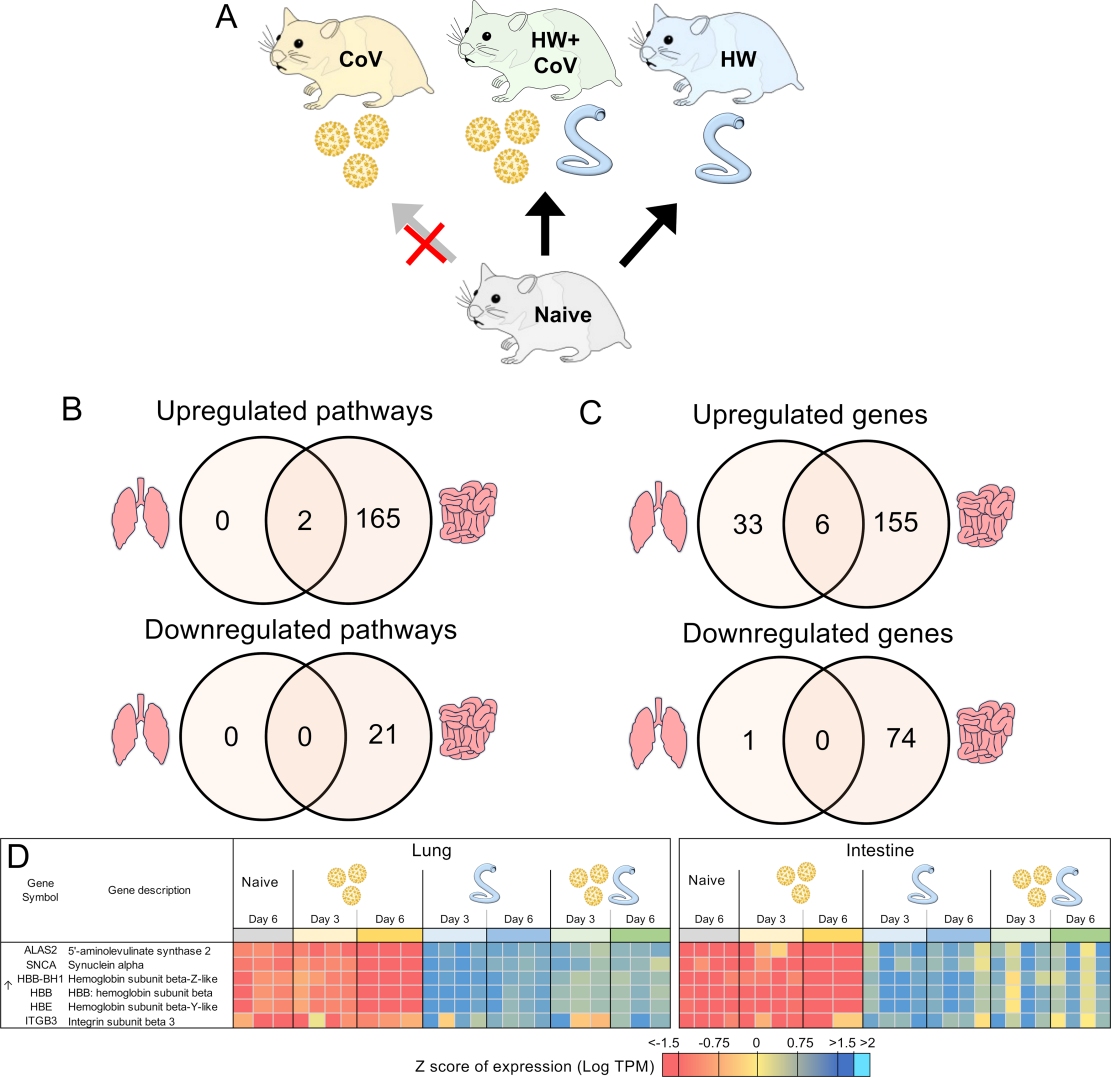
Genes differentially regulated by hookworm but not by SARS-CoV-2, regardless of coinfection status. **(A)** Schematic of the cohorts being compared: The comparison indicated by the red X must not be significant in order for genes to be considered. **(B)** Intersections of pathways of interest enriched among sets of DEGs in both the lung (left) and the intestine (right), at both day 3 and day 6. **(C)** Intersections of DEGs in both the lung and the intestine, at both day 3 and day 6. **(D)** Heatmap representation of the expression levels of gene sets from the intersections of the Venn diagrams from panel **(C)** Genes are sorted by FDR-adjusted *P* values.

After RNA-seq samples were sequenced, the downloaded reads were trimmed for length and adapters using Trimmomatic v0.36 (22). Then, the reads were aligned to the golden hamster genome assembly [MesAur1.0, downloaded from ENSEMBL (23)] using the STAR aligner (24) (2-pass mode, basic). Of the fifty-six samples, six RNA-seq samples failed QC and were excluded from analysis. Samples sizes were N = 4 for 9 groups, N = 3 for 4 groups, and N = 2 for the day 6, CoV intestine group. Significantly differentially expressed genes between each infected cohort compared to the uninfected cohort was identified using DESeq2 (version 1.12.3) (25) with default settings, using scripts previously provided on Protocol Exchange (26). Significantly differentially expressed genes (DEGs) were identified based on an FDR-adjusted *P* value ≤ 0.01, a minimum 2-fold change, and expression detected in at least two samples in the higher-expressed group. Additional analysis between infected cohorts was performed at either 3 or 6 days, but not between days of infection. Differentially expressed gene sets compared to the naive cohort were compared across the different infection cohorts, identifying infection-specific and infection-conserved genes and genes differentially expressed only with coinfection with both the worm and the virus across both time points (3 and 6 days after virus infection). Sample metadata and corresponding NCBI SRA accessions (27) are provided in **Supplementary Table S1**, and complete expression data and differential expression statistics for all genes in all comparisons are provided in **Supplementary Table S2**. Heatmaps were calculated and visualized in MS Excel, and swarm plots were generated using GraphPad PRISM. Some components of **Figures 1**–**7** were generated in BioRender (citation Rosa, B. 2025 https://BioRender.com/jwe4naf). Sets of differentially expressed genes (DEGs) were compared and filtered using MS Excel.

Genes from the hamster genome were assigned putative functional annotations using KEGG (28) and InterProScan (29), and orthology to human genes was identified using annotations from ENSEMBL (23). Pathway enrichment was performed with ShinyGO 0.80 (30), using ENSEMBL (23) annotations for gene ontology biological process terms (31) and KEGG pathway enrichment (28). For all enrichment testing, FDR-adjusted *P* values ≤ 0.05 were used to identify significant enrichment.

### 2.7 CIBERSORTx estimation of relative leukocyte cell abundance

Reciprocal best-hit BLAST (32) (NCBI blastp, v2.7.1+) orthologs between hamster proteins and human proteins were identified based on Ensembl genome annotations [MesAur1.0 and GRCh38.112, respectively (23)]. The relative gene expression values of these 1:1 orthologs were used to estimate the relative abundance of 22 leukocyte cell types by performing gene expression deconvolution with the CIBERSORTx (33) algorithm, using the validated “LM22” leukocyte gene signature matrix (34).

## 3 Results and discussion

This study investigated the impact of prior worm infection on SARS-CoV-2 infection in a Syrian hamster model. Syrian hamsters are considered one of the best animal models for SARS-CoV-2 due to susceptibility to virus infection, ability to transmit the virus, and similarities in disease progression, virus tropism, and severity to humans (35, 36). Syrian hamsters are also susceptible to the hookworm (*Ancylostoma ceylanicum*), which also infects humans as well as other mammals such as dogs and cats. To assess the impact of hookworm infection on SARS-CoV-2 infection and disease, eight groups of age-matched 5–6 week old male hamsters were infected under different conditions and sacrificed at two timepoints each (three and six days post SARS-CoV-2 infection; **Figure 1A**) with four infection regimes: (i) Gavaged with PBS (uninfected; Naive), (ii) Gavaged with 80–100 L3 *A. ceylanicum* larvae, and inoculated with PBS 20 days later (hookworm-infected, HW), (iii) Gavaged with PBS and inoculated with 10,000 infectious units of SARS-CoV-2 (WA1/2020) 20 days later (SARS-CoV-2 infected; CoV) and (iv) Gavaged with 80–100 L3 *Ancylostoma ceylanicum* larvae and inoculated with SARS-CoV-2–20 days later (“coinfected”; HW+CoV).

Six days after SARS-CoV-2 infection, naive hamsters increased in weight by an average of 5.8% of their starting weight compared to before infection, while HW caused a decrease in body weight of 1.9% (*P* = 0.034; **Figure 1B**). SARS-CoV-2 and coinfection resulted in a 15.0% and 16.5% decrease, respectively (*P* = 0.0003 and *P* = 0.0002), similar to weight loss reported in previous SARS-CoV-2-infected Syrian hamsters of similar age [e.g. 7.9 to 15.4% (35), 10 to 17% (37), 4 to 15% (38)]. In the lungs of CoV and HW+CoV hamsters, high viral RNA titers were detected on day 3 (~8.9×10^7^ PFU/mL and 1.0×10^8^ for CoV and HW+CoV groups respectively) and substantially reduced by day 6 post infection (1.0×10^4^ and 5.6×10^4^ PFU/mL; **Figure 1C**), with no significant difference observed between the two groups. Similar results were obtained for the nasal turbinates (day 3: 1.4×10^7^ PFU/mL for CoV and 1.0×10^7^ PFU/mL for HW+CoV; day 6: 1.4x10^3^ PFU/mL for CoV and 1.3x10^3^ PFU/mL for HW+CoV; **Figure 1D**), also in agreement with previously-published studies (e.g. ~1×10^7^ at day 3, ~1×10^3^ at day 6 (35, 39)).

Despite no changes in weight loss or virus titers between the CoV and HW+CoV hamsters, differences in host responses to the infection may still occur. To assess the impact of hookworm infection on the host response to SARS-CoV-2 infection (and vice versa), RNA was extracted from the lungs and intestine of each hamster at the timepoint coinciding with 3 or 6 days post-infection with SARS-CoV-2 (**Figure 1A**), and RNA-seq datasets were generated by Illumina sequencing, generating an average of 55.8 million read pairs per sample (sample metadata and corresponding NCBI SRA accessions (27) provided in **Supplementary Table S1**). Samples clustered according to their overall transcriptome profile generally by cohort according to principal component analysis (PCA), for both the lung (**Figure 2A**) and the intestine samples (**Figure 2B**). Differential gene expression analysis was performed using DESeq2 (25), with significantly differentially expressed genes (DEGs) requiring an FDR-adjusted *P* value ≤ 0.01, a minimum 2-fold change, and expression detected in at least two samples in the higher-expressed group. **Supplementary Table S2** contains lists of DEGs, relative abundance, and differential expression statistics for all genes and all comparisons, which include each infected cohort vs the naive cohort in the same tissue and the infected cohorts compared to each other in the same tissue on the same day.

Following SARS-CoV-2 infection (CoV cohort), 1,700 DEGs were consistently (day 3 and day 6) upregulated (**Figure 2C**), and 311 were downregulated in the lung, compared to only 7 and 22 DEGs (respectively) in the intestine (**Figure 2D**), which may be expected since CoV primarily infects the lungs, with very low loads in the intestine that are probably cleared up by day 6 (40). Likewise, HW only infects the intestine and results in more consistent DEGs in the intestine (217 upregulated, 152 downregulated) compared to the lungs (171 and 4, respectively). With HW+CoV coinfection, 1,053 DEGs were upregulated, and 154 were downregulated in the lung compared to 669 and 559 (respectively) in the intestine. In the lungs, differential gene expression counts were higher at day 6 for CoV-infected cohorts, but higher at day 3 in the HW cohort (**Figure 2C**), while in the intestine, more genes were differentially expressed at day 3 than at day 6 in all infection cohorts (**Figure 2D**).

Pathway enrichment was performed with ShinyGO 0.80 (30) for each set of DEGs to identify significantly enriched gene ontology biological process terms (31) and KEGG pathways (28). The number of significantly enriched pathways among the sets of DEGs compared to the naive controls (as described above) are summarized in **Supplementary Figure S1**. As expected, in the lungs, the “Coronavirus disease-COVID-19” KEGG pathway (maua05171) was significantly enriched among DEGs upregulated in HW+CoV vs both naive and HW at both days (*P* ≤ 1.8x10^–5^ in all four comparisons), but not vs the CoV cohort. However, no pathways were significantly enriched in the lungs among all three sets of DEGs as outlined in **Figure 3A**. In the subsections below, both specific differentially expressed genes and pathway enrichment among the larger sets of DEGs will be described in each comparison in order to thoroughly explore the results.

Counts of DEGs and the number of enriched pathways for each set of DEGs are provided in **Supplementary Table S3**, and FDR-adjusted *P* values for significance for all enriched pathways in all sets of DEGs are provided in **Supplementary Table S4**, with parsed pathways of interest provided in **Supplementary Table S5**. Details of sets of DEGs and enriched pathways are described in the sections below.

### 3.1 Genes uniquely upregulated in the lungs of coinfected hamsters at day 6 are associated with B cell immunity

To identify coinfection-specific gene transcriptional signatures, we identified DEGs in the HW+CoV cohort relative to the other three cohorts (naive, CoV, and HW; **Figure 3A**) at day 3 and day 6 in the lungs and in the intestine, ensuring that differential expression was very specific to coinfection as opposed to single CoV or HW infections. DEGs were categorized by differential regulation direction (higher/upregulated or lower/downregulated), time point (day 3 or 6) and tissue (lung or intestine).

Five DEGs were significantly higher in the lungs of the HW+CoV cohort compared to each of the other cohorts on day 6 (**Figure 3B**). These included *RBM38* (RNA binding motif protein 38), which was significantly upregulated at day 6 in both individual infections but even more significantly upregulated at day 6 in the coinfection cohort. *RBM38* is essential for proper hematopoiesis (41) and belongs to a class of proteins that includes RMB24, which inhibits the translation of SARS-CoV-proteins by targeting single or double-stranded regions of RNA molecules (42). Three of these genes were not upregulated by day 6 in either of the single infections: (i) *ATF5* (Activating transcription factor 5), which regulates B cell survival during stress (43) and has roles in cellular differentiation (44); (ii) *IGHM* (Immunoglobulin heavy constant mu), which plays an important role in the neutralization of SARS-CoV2 (45); and (iii) *PRDX2* (Peroxiredoxin 2), which serves as the primary antioxidant in erythrocytes, effectively neutralizing hydrogen peroxide generated endogenously through hemoglobin autoxidation (46). The extent of PRDX2 oxidation is notably elevated in various pathological conditions, making it a potential biomarker for oxidative stress. Finally, *JCHAIN* (Joining chain of multimeric IgA and IgM) was significantly upregulated in the lungs of HW but not the CoV hamsters at day 6, but was far more significantly upregulated in the HW+CoV cohort (**Figure 3C**). JCHAIN regulates the multimerization of secretory IgM and IgA and is required for their transport across the mucosal epithelium (47, 48). Immunoglobulin-A (IgA), the “mucosal Immunoglobulin,” has been a focal point in the study of SARS-CoV-2 immunity, diagnosis, and immunization (49).

Overall, the upregulation of these specific genes at day 6 indicates that coinfection with HW+CoV leads to an increase in hematopoiesis (*RBM38*) and B cells (*ATF5*), as well as an increase in *IgM* and *JCHAIN* which are both produced and secreted by B cells, with important roles in COVID-19 immunity. These results suggest that pre-infection with hookworm may produce a more robust B cell response in the lungs of infected animals by day 6.

### 3.2 Genes uniquely differentially regulated in the intestine of coinfected hamsters are associated with inflammation

Several pathways were significantly enriched in the intestine among all three sets of coinfection DEGs, as outlined in **Figure 3A** (pathways listed in **Supplementary Table S5A**). On day 3, 11 GO terms related to immune responses and 2 related to stress responses were significantly enriched among upregulated DEGs, and 3 terms related to lipid transport were enriched among downregulated DEGs. This indicates an increase in overall immune and stress response activations corresponding with lipid metabolism downregulation in the intestine due to coinfection compared to single infections. On day 6, overall metabolic pathways and specific metabolism of retinol, xenobiotics, ascorbate, and aldarate were downregulated.

Two genes were significantly upregulated in the intestines of the coinfection cohort compared to all other sample groups in the intestine on day 6, and both were not significantly upregulated at day 6 in either of the single infections: (i) *FOLR2* (Folate receptor 2; **Figure 3D**), which is a glycosylphosphatidylinositol-anchored glycoprotein that is predominantly expressed by monocytes and macrophages (50). *FOLR2* isoform FRβ is upregulated in activated macrophages during acute inflammation resulting from the pathogenesis of various human inflammatory diseases, suggesting a role in this receptor in macrophage-controlled inflammation regulation (50). This is consistent with research showing a role for folate in regulating inflammatory responses including those induced by pathogen-associated molecular patterns (PAMPs) such as lipopolysaccharide (LPS) (50–52); (ii) *PLA2G4F* (Phospholipase A2 group IVF, *cPLA2ζ*), which has a primary known role in mediating arachidonic acid release (53) including from fibroblasts (54) ([which play significant roles in tissue maintenance and immune homeostasis during inflammation and damage in the intestine (55)]. Arachidonic acid and its derivatives play a critical role in inducing inflammation in the intestine, including in diseases such as Crohn’s disease and ulcerative colitis (56), which suggests a possible mechanism for PLA2G4F regulation of intestinal inflammation. Another Phospholipase A2 family gene, *sPLA2-IB*, is upregulated by intestinal epithelial cells during nematode infection, and is necessary for their expulsion from the intestine due to its activity in hydrolyzing worm phospholipids (57). While no such activity has been demonstrated for PLA2G4F, this suggests other possible mechanisms for Phospholipase A2 activity against pathogenic infection in the intestine. The upregulation of both *FOLR2* and *PLA2G4F* in the intestine in response to HW+CoV may suggest a mechanism for increasing intestinal inflammation.

One gene, *FABP3* (Fatty acid binding protein 3), was significantly downregulated in the intestines of both the single infections but was much more significantly downregulated in the coinfection cohort compared to all other sample groups in the intestine on day 3 (**Figure 3E**). *FABP3* is a negative regulator of inflammation in the skin, so its significant downregulation in this dataset may be linked to increased inflammation in the intestine (58). However, FABP3 is typically studied for its roles in heart muscle tissue, while FABP2 is more typically described in the intestine, with SARS-CoV-2 infection having been shown to reduce circulating FABP2, correlating with systemic inflammation (59). In that study, *FABP3* followed a similar expression to *FABP2* but did not reach significance in the same comparisons.

Three genes were significantly lower in the coinfection cohort’s intestines than all other sample groups in the intestine on Day 6. The first, *UGT2A3* (UDP-glucuronosyltransferase 2A3), was significantly downregulated at day 6 in the HW cohort but was much more significantly downregulated in the coinfected cohort. UGT2A3 is not well-characterized but has been shown to deactivate bacterial-derived intestinal hyodeoxycholic acid (60), which has been shown to attenuate inflammation by targeting the TLR4/MD2 complex (61). The other two genes in this set were not significantly downregulated in either of the single infections: (i) *SLC13A1* (Solute carrier family 13 member 1; *NAS1*), which mediates sulfate absorption across small intestinal epithelia (62) (**Figure 3F**). *NAS1* knockout mice with subsequent intestinal sulfomucin content showed enhanced intestinal permeability and enhanced DSS-induced colitis (63), so its downregulation may be associated with increased intestinal inflammation as well; (ii) *CES1D* (Carboxylesterase 1D-like), which increases LPS-induced inflammation in the lungs (64), and which is primarily involved in lipid metabolism (65). In the intestine, lipid mediators derived from polyunsaturated fatty acids play complex roles in inflammatory processes (66), so the downregulation of *CES1D* may indirectly affect inflammation in a similar manner to UGT2A2 and hyodeoxycholic acid.

Taken together, the differential regulation of intestinal genes, specifically with coinfection, may reflect causes of or the responses to intestinal inflammation, indicative of inflammatory reactions in the gut of coinfected hamsters, and were not observed in hamsters infected with either pathogen alone.

### 3.3 Genes consistently upregulated in the lungs only in SARS-CoV-2-infected hamsters reveal immune responses attenuated by pre-infection with hookworm.

Several gene sets were identified that were differentially expressed in the lungs of the CoV cohort compared to coinfected (HW+CoV) hamsters (**Figures 4A, B**). Many of these genes (e.g., **Figures 4C–E**) were strongly upregulated by CoV alone but showed significantly weaker upregulation in the HW+CoV cohort, suggesting that hookworm coinfection attenuated these responses. In other cases (e.g., **Figure 4F**), genes that were downregulated by CoV were upregulated in HW+CoV, but significantly less upregulated than HW alone.

Three genes were significantly higher in the lungs of the CoV cohort compared to each of the other cohorts on day 3, representing an attenuation of the upregulation by CoV resulting from pre-infection with HW: (i) *FCGR4* (Low-affinity immunoglobulin gamma Fc region receptor III-like; **Figure 4C**), the mouse ortholog of human *FCGR3A*/*CD16* (67), which is the major Fc receptor that mediates uptake of opsonized SARS-CoV-2 in monocytes (68). In individuals infected with the human hookworm *Necator americanus*, *FCGR3A* was significantly lower in dendritic cells collected from the blood (69), which may explain its attenuation in coinfected lung samples. Higher *FCGR3A* expression is also associated with recovery from COVID, compared to Long COVID patients (70); (ii) *CTSK* (Cathepsin K), which is capable of cleaving SARS-CoV-2 spike protein (71) and when transgenically overexpressed in mouse lungs, is associated with reduced collagen deposition, improved lung function parameters, and resistance to pulmonary fibrosis (72). CTSK also contributes to intestinal homeostasis and tissue architecture, with its reduction being associated with higher collagen IV and laminin in the mouse intestine, suggesting a potential interaction with hookworm infection (73); (iii) *CHIL1* (Chitinase 3 like 1; *CHI3L1*; **Figure 4D**), which is associated with poorer prognosis for patients with SARS-CoV-2 infection (74), and may exacerbate associated lung damage and inflammation by stimulating ACE2 and proteases that prime the viral spike SARS-CoV-2 protein (75). Impaired TH2 responses were observed in *CHI3L1* knockout (-/-) mice infected with the intestinal helminth *Heligmosomoides polygyrus* (76). In the intestine, CHI3L1 is secreted from colonic epithelial cells and its expression exacerbates intestinal inflammation (77), but hookworm infection, conversely, is associated with a reduction of intestinal inflammation, and is considered a potential therapeutic for a range of inflammatory diseases (78). This may suggest that the attenuation of *CHI3L1* expression in SARS-CoV-2-infected lungs of coinfected hamsters may reflect the anti-inflammatory effects of hookworm infection, which may lead to better SARS-CoV-2 outcomes.

On day 3 in the lungs, two related KEGG pathways were significantly enriched among the three sets of DEGs in **Figure 4A**: Rheumatoid arthritis (maua05323) and Toll-like receptor (TLR) signaling pathway (maua04620; **Supplementary Table S5B**); TLRs contributes to SARS-CoV-2 clearance and disease resolution, with important roles in inflammatory regulation (79).

At day 6, seven genes were significantly higher in the lungs of the CoV cohort compared to all other sample groups: (i) *HMOX1* (Heme oxygenase 1; HO-1; **Figure 4E**), which confers protection from inflammatory conditions through the removal of heme in tissues including the lungs (80), and has been suggested as an important predictor of COVID-19 severity (81). Upregulation of *HMOX1* has been suggested as a promising target for the treatment and prevention of SARS-CoV-2 (82); (ii) Granzyme C-like, which is predominantly synthesized by mouse CD4+ and CD8+ T cells under *in vitro* conditions or during mixed lymphocyte reactions (83). Granzymes including the human ortholog of Granzyme C-like (Granzyme B) have the capability to directly inhibit viruses by inducing proteolysis of viral or host cell proteins necessary for the viral entry, release, or intracellular trafficking, and by augmenting pro-inflammatory antiviral cytokine responses (84); (iii) *CD209C* (Cluster of Differentiation 209; also called Dendritic Cell-Specific Intercellular Adhesion Molecule-3-Grabbing Non-integrin, *DC-SIGN*), is a type C lectin found on alveolar macrophages and dendritic cells, which plays a key role in innate immunity and antiviral defense by acting both as a pathogen recognition receptor and a cell adhesion receptor (85). It can bind various microorganisms including SARS-CoV-2, and its polymorphisms are significantly associated with an increased risk of COVID-19 infection (85); (iv) *KRT14* (Keratin, type I cytoskeletal 14), the expression of which can serve as a marker for lung regeneration and repair during alveolar damage (86); (v) *LAD1* (Ladinin-1; Leukocyte adhesion deficiency-1), which is expressed in squamous and glandular epithelia, with a well-established function as an anchoring filament protein. LAD-1 plays a critical role as a component of the basement membrane zone and is negatively correlated in lung cancer survival (87); (vi) *FST* (Follistatin), which is associated with COVID-19 mortality (88) and severe Long Covid (89) and (vii) *PEX10* (Peroxisomal biogenesis factor 10), which is critical for peroxisomal matrix-protein import (90), but has no previously described associations with SARS-CoV-2 infection.

Overall, these data indicate that certain host genes and pathways strongly upregulated by CoV infection become attenuated or even reversed when hamsters are pre-infected with HW. The DEGs include many known biomarkers of COVID-19 severity and outcomes, highlighting the importance of considering coinfections when evaluating transcriptional signatures of infection. The enrichment of TLRs additionally highlights the capacity of hookworm infection to reshape the antiviral inflammatory response. Consequently, hookworm-induced immune regulation may reduce lung inflammation and potentially improve SARS-CoV-2 outcomes, pointing to broader implications for helminth-viral coinfections.

### 3.4 Genes with attenuated or reversed SARS-CoV-2-induced downregulation in the lung during coinfection with hookworm include enrichment of hemoglobin b genes

A total of 47 genes were significantly lower in the lungs the CoV cohort compared to all other sample groups in the lung at day 6 (The top five are shown in **Figure 4B**), indicating attenuated downregulation responses with in the HW+CoV cohort. All four Syrian hamster hemoglobin b genes were among these 47 (*HBB-BH1* [**Figure 4F**], *HBE*, *HBB-Y*, and *HBB*; FDR-adjusted enrichment *P* = 1.8x10–^7^ for IPR002337). SARS-CoV-2 binds to red blood cell progenitors and dysregulates hemoglobin metabolism (91), but HW infection is known to be negatively associated with Hb concentrations in the blood (92), likely due to their blood feeding, so increased expression of these genes may indicate compensation by the host to regenerate Hb. The genes among this group that were the most significantly downregulated by CoV at day 6 also included *AOC3* (Amine Oxidase Copper Containing 3; *VAP-1*), which is dramatically increased in expression in severe COVID-19 cases but downregulated during the convalescent stage (93) and *IGFBP3* (Insulin-like growth factor binding protein 3), which was downregulated in severe and critical COVID-19 patients (94). The 47 genes were significantly enriched for oxygen transport (GO:0015671, *P* = 3.3x10^-6^), possibly due to adaptations to HW-induced anemia.

### 3.5 Genes with reversed hookworm-induced downregulation in the intestine during coinfection with SARS-CoV-2 are both related to Type I interferon

Two genes were significantly lower in the intestines of the HW-only cohort compared to all other sample groups at day 3 (**Figure 5A**): (i) *TAP1* (Transporter 1, ATP binding cassette subfamily B member; **Figure 5B**) is a membrane-bound protein composed of two membrane-spanning domains and belongs to the ATP-binding cassette (ABC) transporter superfamily, playing a critical role in transporting antigens from the cytoplasm to the endoplasmic reticulum, where it interacts with major histocompatibility complex (MHC) class I molecules (95) and (ii) *IRF7* (Interferon regulatory factor 7; **Figure 5C**), which is recognized as the master regulator of type I IFN production (96). Previously, the intestinal helminth *H. polygyrus* has been shown to modulate RSV lung inflammation and disease through the induction of type I IFN responses mediated through the gut microbiota (10). TAP1 also demonstrates broadly antiviral activities through the activation of type I IFN responses, including IRF3 (97), so these two genes may indicate a shared type I IFN mechanism that is downregulated by HW infection but upregulated during HW+CoV due to the influence of CoV infection on intestine transcription.

### 3.6 Covid-upregulated genes that are not upregulated by hookworm infection follow expectations for immune responses to COVID-19 infection

In addition to the genes uniquely differentially regulated during coinfection described above, to characterize general SARS-CoV-2 responses, we also identified DEGs consistently differentially regulated by CoV but not by HW (**Figure 6A**). As expected, many more pathways were significantly enriched among the DEGs in the lungs (**Figure 6B**), with the top most significantly enriched pathway among lung-upregulated gene sets being “Coronavirus disease-COVID-19” (maua05171; FDR-adjusted *P* < 1.9x10–^5^ at day 3 and *P* < 10–^35^ at day 6), and the other top enriched pathways being related to the immune system, inflammatory responses and cytokine production (**Supplementary Table S5D**). In both the lung and the intestine, 8 pathways were significantly enriched among upregulated DEGs in both CoV comparisons, including “cellular response to cytokine stimulus”, “response to interferon-beta,” and “NOD-like receptor signaling pathway”, while 6 terms, including several terms related to type I interferon were only significantly enriched among the intestine-upregulated DEGs but not the lungs (**Supplementary Table S5D**). No terms overlapped both tissues among downregulated DEGs (**Figure 6B**).

In the lungs, there were 324 DEGs upregulated by SARS-CoV-2 both in the CoV and the HW+CoV cohorts, which were not upregulated by HW alone on both days. Because of the low number of genes differentially regulated by CoV in the intestine at day 6 (**Figure 2D**), the same parsing was performed using only day 3, identifying 72 CoV-specific upregulated DEGs. There were 33 DEGs overlapping these two gene sets between the two tissues, representing systemic upregulated genes spanning tissues (**Figure 6C**, where the probability of this much overlap is *P* < 10^–15^ by random chance; binomial distribution test). These 33 genes were enriched for many pathways and functions (**Table 1**), including 17 genes assigned to “response to biotic stimulus” (GO:009607, FDR-adjusted *P* = 3.6x10^-13^) and 6 genes assigned to the “Coronavirus disease-COVID-19” KEGG pathway (maua05171, FDR-adjusted *P* = 1.4x10^-5^). In order of average DESeq2 significance, these six genes are (i) *MX2*, an IFN-stimulated gene with broad antiviral activity (98), which correlates with SARS-CoV-2 viral load (99), (ii) *OAS1*, which protects against SARS-CoV-2 infections via RNase L enzyme activity in humans, and protects against viral infections through non-canonical IFN pathways in both mouse and human (100), (iii) *EIF2AK2*, which is important for activating IFN responses and interfering with host cell translation during SARS‐CoV‐2 infection (101), (iv) *STAT1*, which plays a crucial role in viral infection control, including for SARS-CoV-2 (102), (v) ADAR, which protects against SARS-CoV-2 infection through enhanced T cell responses and reduced viral load (103), and (vi) *STAT2*, which restricts SARS-CoV-2 dissemination but can drive severe pneumonia in hamsters (104). Other genes among the 33 include interferon-related genes *IFIT3*, *IFI35* and *IFI44*. The complete list of 33 genes and their relative expression across both intestine and lung samples are shown in **Figure 6D**, and highlights the value of the overall dataset in identifying known SARS-CoV-2 responsive DEGs.

**Table 1:** Significantly enriched Gene ontology terms and KEGG pathways among the 33 genes consistently upregulated by SARS-CoV-2 infection (alone or during coinfection), but not by hookworm alone, in both the lung and the intestine.

Database	Pathway id	Description	Total term size	# in gene set	FDR-adjusted *P*
Gene Ontology (BP) (Top 15 shown)	GO:0009607	Response to biotic stimulus	757	17	3.5×10^-13^
	GO:0051707	Response to other organism	727	17	3.5×10^-13^
	GO:0098542	Defense response to other organism	495	15	3.5×10^-13^
	GO:0044419	Biological proc. involved in interspecies interaction	814	17	9.2×10^-13^
	GO:0009615	Response to virus	217	11	1.1×10^-11^
	GO:0051607	Defense response to virus	156	10	1.3×10^-11^
	GO:0140546	Defense response to symbiont	157	10	1.3×10^-11^
	GO:0045087	Innate immune response	300	11	2.5×10^-10^
	GO:0006952	Defense response	847	15	3.5×10^-10^
	GO:0034097	Response to cytokine	488	12	1.7×10^-9^
	GO:0060337	Type I interferon signaling pathway	33	6	1.7×10^-9^
	GO:0071357	Cellular response to type I interferon	34	6	1.8×10^-9^
	GO:0002831	Reg. of response to biotic stimulus	214	9	6.0×10^-9^
	GO:0009605	Response to external stimulus	1553	17	9.6×10^-9^
	GO:0060338	Reg. of type I interferon-mediated signaling pathway	28	5	6.6×10^-8^
KEGG	maua05162	Measles	101	6	9.9×10^-7^
	maua05164	Influenza A	121	6	1.4×10^-6^
	maua05171	Coronavirus disease-COVID-19	190	6	1.4×10^-5^
	maua05160	Hepatitis C	119	5	2.2×10^-5^
	maua04217	Necroptosis	87	4	1.4×10^-4^
	maua04621	NOD-like receptor signaling pathway	114	4	3.5×10^-4^
	maua05165	Human papillomavirus infection	241	5	4.0×10^-4^
	maua05169	Epstein-Barr virus infection	148	4	7.2×10^-4^
	maua05168	Herpes simplex virus 1 infection	200	4	2.0×10^-3^
	maua05167	Kaposi sarcoma-associated herpesvirus infection	137	3	7.8×10^-3^
	maua04623	Cytosolic DNA-sensing pathway	41	2	9.3×10^-3^
	maua04625	C-type lectin receptor signaling pathway	82	2	0.033
	maua04380	Osteoclast differentiation	91	2	0.037
	maua04120	Ubiquitin mediated proteolysis	95	2	0.037
	maua04630	JAK-STAT signaling pathway	102	2	0.040
	maua05161	Hepatitis B	113	2	0.045

Considering the downregulated DEGs with the same intersection of differential expression across cohorts, we identified 107 lung-downregulated genes and 19 intestine-downregulated genes, with the only intersection between the tissues being the zinc ion transporter gene *SLC39A4* (**Figure 6C**), which may be involved in accelerating tumor development by impairing immune responses in cytokine signaling pathways and IL-17 signaling pathways (105).

### 3.7 Hookworm-upregulated genes that are not upregulated by SARS-CoV-2 are associated with hemoglobin

Like the analysis in the previous section, we also identified DEGs that were consistently differentially regulated by HW on both days but not by CoV infection alone (**Figure 7A**). Only two pathways were consistently significantly enriched in the lungs among the upregulated DEGs (gas transport and oxygen transport), and both of these were also significantly enriched among the upregulated DEGs in the intestine (**Supplementary Table S5E**), likely compensating for hookworm-induced anemia (106). An additional 167 pathways were enriched among the DEGs upregulated in each comparison in the intestine, the most significant of which were related to cell motility, blood vessel development, and leukocyte activation. Among the DEGs downregulated in each comparison in the intestine, 12 pathways were enriched including pathways related to anion transport, bile secretion and lipid catabolism (**Supplementary Table S5E**).

In the lungs, 39 DEGs were upregulated by hookworm on both days, both in the HW infection and the HW+CoV cohorts, which were not upregulated by CoV infection alone (**Figure 7B**). In the intestine, the same parsing identified 161 HW-specific upregulated genes. There were 6 genes overlapping these two gene sets between the two tissues (**Figure 7C**), representing systemic upregulated genes spanning tissues (where the probability of this much overlap is 5.0x10^–8^ by random chance). In order of average DESeq significance, these 6 genes were *ALAS2*, *SNCA*, three hemoglobin b genes (*HBB-BH1*, *HBB*, *HBE*), and *ITGB3* (**Figure 7D**). The hemoglobin genes were described above in relation to genes with attenuated or reversed CoV-induced downregulation during coinfection with HW+CoV, highlighting the worm-induced systemic upregulation of these genes despite their significant downregulation in the CoV-only cohorts. The precursor protein of ALAS2 includes motifs responsive to heme, which hinder its translocation into mitochondria upon heme binding (107), and erythropoietic factors and iron availability are the principal regulators influencing ALAS2 (107, 108), so there may be a link to heme with that gene as well.

Among the HW-downregulated genes, 74 were consistently downregulated by HW but not CoV in the intestine, but only one (*PLK2*; Polo-like kinase2) was consistently downregulated in the lungs. Genetic deletion of *PLK2* in the lungs induces a pro-fibrotic phenotype, suggesting a role in regulating fibrosis (109).

### 3.8 CIBERSORTx-estimated relative abundance of leukocyte cells in lung samples

The relative gene expression profiles for reciprocal best-hit (1:1) orthologs between hamster proteins and human were used to estimate the relative abundance of 22 leukocyte cell types in the lung samples, using CIBERSORTx (33) (see Methods; **Supplementary Table S2**; **Supplementary Table S6**). Both CoV and HW+CoV infection resulted in a significant increase in activated natural killer (NK) cells (from 12.6% to more than 21.1% at both days), but these were significantly reduced with HW alone (8.3%); In previous research, NK cell tissue distribution was found to be affected by CoV infection, and a prompt NK cell response was found to possibly determine a good clinical outcomes (110). M0 macrophages were significantly reduced by both CoV and HW+CoV (from 5.7% to ≤ 1% each), while M1 and M2 macrophages were significantly increased in both of these cohorts [which produce pyrogenic and inflammatory mediators in response to CoV infection (111)]. Additionally, resting mast cells (7.3% in naive) were significantly reduced by CoV and HW+CoV (≤1% each), while activated mast cells increased but not significantly in these cohorts; Activation of mast cells is a potential biomarker for CoV infection, and activation profiles are associated with severe outcomes (112). Relative to CoV, only memory B cells were significantly higher in HW+CoV, at day 3 (2.6% vs 0.4%; FDR-corrected *P* = 0.021). Overall, these results suggest that, with the exception of memory B cells, leukocyte cell recruitment responses to CoV are not substantially affected by prior HW infection, despite the differential activation of many genes and pathways as described above.

## 4 Conclusion

Our study provides insights into how concurrent hookworm and SARS-CoV-2 infections affect host transcriptional responses across the lung and intestine, shedding light on potential immunological interactions between these pathogens. Our dataset included RNA-seq samples generated from lung and intestinal tissue from cohorts of naive, CoV-infected, HW-infected, and HW+CoV hamsters collected at two-time points (3 days and 6 days post-infection with CoV). Each of the infected cohorts showed significantly reduced weight compared to naive, and virus titers were similar for CoV and HW+CoV cohorts, lowering by several orders of magnitude between day 3 and day 6. This experimental design facilitated several strategies for intersecting DEG sets of interest to analyze different aspects of the host response to infection.

Consistent transcriptional differences were observed in the coinfection cohort compared to naive and single-infection cohorts, underscoring how helminth-induced immunomodulation can reshape the host response to viral infections. Coinfected hamsters showed distinct transcriptional signatures in the lungs by day 6, including the upregulation of *ATF5*, *IGHM*, and *JCHAIN*, which together with the increase in memory B cells, suggest that prior hookworm infection may enhance B cell-mediated immunity and secretory IgM/IgA pathways in the lungs, to rapidly clear the virus. Hookworm infection is known to induce robust Th2 responses driven by IL‐4 (6, 113, 114).

In the intestine, coinfection resulted in the distinct upregulation of multiple inflammatory and stress-related gene signatures, including *FOLR2* and *PLA2G4F*, which were not significantly upregulated in the single-infection cohorts. These and other coinfection-specific genes point to an intensified inflammatory state in the gut, possibly reflecting differences in the hookworm’s capacity to modulate inflammatory processes during overlapping viral challenges. Pathway enrichment analysis of each set of DEGs also supported the upregulation of stress responses and the downregulation of lipid metabolism pathways in the intestine.

An additional analysis identified several host genes and pathways, including *FCGR4*, *CTSK*, *HMOX1*, and *Granzyme C*, that are strongly differentially regulated by CoV infection alone and become attenuated or even reversed when hamsters are pre-infected with HW. These shifts in gene expression, including overall enrichment in toll-like receptor signaling, indicate specific targets and pathways that are predisposed to differential responses to CoV with prior HW infection, potentially indicating mechanisms for reduced inflammation and improved CoV infection outcomes. Among the genes with attenuated CoV upregulation when coinfected with HW were previously proposed host protein biomarkers for the prognosis of COVID severity [*CHI3L1* (74), *HMOX1* (82)], Long COVID [*FCG4*/*FCGR3A* (70) and *FST* (89)] and mortality [*FST* (88)]; These may indicate key genes involved in the lower COVID outcomes in helminth-endemic regions (12).

Similarly, HW infection suppresses select type I interferon-related genes (*TAP1* and *IRF7*) in the intestine, but this effect is reversed by CoV coinfection, suggesting a shared IFN-driven antiviral mechanism. In the lungs, DEGs associated with HW-only infection are consistently enriched for the upregulation of oxygen transport and downregulation of many immune system processes, including cytokine production, but these pathways are no longer enriched in the HW+CoV cohort, indicating a reversal of HW-induced immunomodulation in response to CoV infection.

Finally, we observed expected genes and enrichment among DEGs associated with CoV but not HW (including enrichment of the “Coronavirus disease-COVID-19” pathway in the lungs, and cytokine, interferon and NOD signaling pathways in both tissues), and many genes and pathways indicative of HW-induced anemia were differential with HW but not CoV. These results highlight the high quality of the dataset by reproducing expected biological findings and provide an additional resource to data mine for the impact of specific infections on the transcriptome of the lung and the intestine.

Overall, by investigating CoV and HW coinfections and comparing them to naive and single infections, we have successfully identified host responses unique to coinfection, analyzed from several perspectives, including the reversal of infection-induced differential regulation. Given the high prevalence of hookworm and other helminth infections in many resource-limited regions, understanding how these parasites influence the course and severity of viral infections, particularly in the respiratory tract, holds significant public health and clinical relevance. Future work, including functional validation of coinfection-specific targets, evaluation of differences of expression at the protein level, mechanistic studies of helminth-associated immune modulation, and experiments with other helminth species with different tropisms, will help clarify whether targeting these pathways can reduce severe outcomes in viral respiratory diseases, especially within populations heavily burdened by parasitic infections.

## Data Availability

The datasets presented in this study can be found in online repositories. The names of the repository/repositories and accession number(s) can be found in the article/**Supplementary Material**.

## Supplementary material

Supplementary Table 1 Metadata, read mapping statistics, and SRA accessions for all RNA-seq samples.

Supplementary Table 2 Functional annotations, DEG lists, fragment counts, relative gene expression levels and DEG statistics for all genes and all DESeq comparisons.

Supplementary Table 3 DEG counts, and significantly enriched pathway counts for all DESeq comparisons.

Supplementary Table 4 FDR-adjusted P values for all significantly enriched pathways among all sets of DEGs.

Supplementary Table 5 Intersections of significantly enriched GO terms and KEGG pathways among sets of differentially expressed genes of interest. Values shown are -Log FDR-adjusted P values for enrichment among the DEG sets indicated.

Supplementary Table 6 Estimated relative abundance of 22 leukocyte cell types in each of the lung RNA-seq samples, deconvoluted using the “LM22” dataset in CIBERSORTx (with 1:1 reciprocal-best orthologs between hamster and human).

Supplementary Figure 1 Counts of significantly enriched Gene Ontology terms + KEGG pathway counts among significantly differentially expressed genes in each infected cohort relative to the uninfected controls, **(A)** in the lungs and **(B)** in the intestine. For all enrichment testing, FDR-adjusted *P* values ≤ 0.05 were used to identify significant enrichment.

Supplementary Figure 2 Average estimated relative proportions of immune cell types, estimated by CIBERSORTx. Significance values represent two-tailed T-tests (unequal variance) compared to the Naive cohort, FDR-corrected for multiple test correction across all tests (* *P* ≤ 0.05, ** *P* ≤ 0.01, *** *P* ≤ 0.001). Relative proportions per sample and exact *P* values are provided in **Supplementary Table S6**. Blue shading = significantly higher, red shading = significantly lower, green shading reflects the relative abundance of each cell type in the naive sample.

## References

[1] World Health Organization. Number of Covid-19 Deaths Reported to Who: WHO COVID-19 dashboard(2025). Available online at: https://data.who.int/dashboards/covid19/deaths (Accessed February 1, 2025).

[2] World Health Organization. Covid-19 Cases, World: WHO COVID-19 dashboard(2025). Available online at: https://data.who.int/dashboards/covid19/cases (Accessed February 1, 2025).

[3] Mueller AL McNamara MS Sinclair DA. Why does covid-19 disproportionately affect older people? Aging (Albany NY). (2020) 12:9959–81. doi: 10.18632/aging.103344, PMID: 32470948 PMC7288963

[4] Rosenke K Adjemian J Munster VJ Marzi A Falzarano D Onyango CO. Plasmodium parasitemia associated with increased survival in ebola virus-infected patients. Clin Infect Dis. (2016) 63:1026–33. doi: 10.1093/cid/ciw452, PMID: 27531847 PMC5036915

[5] Bartsch SM Hotez PJ Asti L Zapf KM Bottazzi ME Diemert DJ. The global economic and health burden of human hookworm infection. PloS Negl Trop Dis. (2016) 10:e0004922. doi: 10.1371/journal.pntd.0004922, PMID: 27607360 PMC5015833

[6] Peng J Federman HG Hernandez CM Siracusa MC. Communication is key: innate immune cells regulate host protection to helminths. Front Immunol. (2022) 13:995432. doi: 10.3389/fimmu.2022.995432, PMID: 36225918 PMC9548658

[7] Schlosser-Brandenburg J Midha A Mugo RM Ndombi EM Gachara G Njomo D. Infection with soil-transmitted helminths and their impact on coinfections. Front Parasitol. (2023) 2:1197956. doi: 10.3389/fpara.2023.1197956, PMID: 39816832 PMC11731630

[8] Desai P Karl CE Ying B Liang CY Garcia-Salum T Santana AC. Intestinal helminth infection impairs vaccine-induced T cell responses and protection against Sars-Cov-2 in mice. Sci Transl Med. (2024) 16:eado1941. doi: 10.1126/scitranslmed.ado1941, PMID: 39167662

[9] Hartmann W Brunn M-L Stetter N Gagliani N Muscate F Stanelle-Bertram S. Helminth infections suppress the efficacy of vaccination against seasonal influenza. Cell Rep. (2019) 29:2243–56.e4. doi: 10.1016/j.celrep.2019.10.051, PMID: 31747598

[10] McFarlane AJ McSorley HJ Davidson DJ Fitch PM Errington C Mackenzie KJ. Enteric Helminth-Induced Type I Interferon Signaling Protects against Pulmonary Virus Infection through Interaction with the Microbiota. J Allergy Clin Immunol. (2017) 140:1068–78 e6. doi: 10.1016/j.jaci.2017.01.016, PMID: 28196762 PMC6485385

[11] Desai P Diamond MS Thackray LB. Helminth-virus interactions: determinants of coinfection outcomes. Gut Microbes. (2021) 13:1961202. doi: 10.1080/19490976.2021.1961202, PMID: 34428107 PMC8405156

[12] Wolday D Gebrecherkos T Arefaine ZG Kiros YK Gebreegzabher A Tasew G. Effect of co-infection with intestinal parasites on covid-19 severity: A prospective observational cohort study. EClinicalMedicine. (2021) 39:101054. doi: 10.1016/j.eclinm.2021.101054, PMID: 34368662 PMC8324426

[13] Al-Kuraishy HM Al-Gareeb AI Alkazmi L El-Bouseary MM Hamad RS Abdelhamid M. The potential nexus between helminths and sars-cov-2 infection: A literature review. J Immunol Res. (2023) 2023:5544819. doi: 10.1155/2023/5544819, PMID: 37383608 PMC10299886

[14] Adjobimey T Meyer J Terkeš V Parcina M Hoerauf A. Helminth antigens differentially modulate the activation of Cd4(+) and Cd8(+) T lymphocytes of convalescent covid-19 patients *in vitro* . BMC Med. (2022) 20:241. doi: 10.1186/s12916-022-02441-x, PMID: 35764965 PMC9241220

[15] Rissmann M Veldhuis Kroeze EJB Tielens AGM Rockx B van Hellemond JJ. Influence of a chronic schistosoma mansoni infection on the outcomes of a sars-cov-2 infection in the hamster model. J Infect. (2023) 87:273–6. doi: 10.1016/j.jinf.2023.07.002, PMID: 37419283

[16] Miguel DC Brioschi MBC Rosa LB Minori K Grazzia N. The impact of covid-19 on neglected parasitic diseases: what to expect? Trends Parasitol. (2021) 37:694–7. doi: 10.1016/j.pt.2021.05.003, PMID: 34059455 PMC8120483

[17] Oyesola OO Hilligan KL Namasivayam S Howard N Clancy CS Zhao M. Exposure to Lung-Migrating Helminth Protects against Murine Sars-Cov-2 Infection through Macrophage-Dependent T Cell Activation. Sci Immunol. (2023) 8:eadf8161. doi: 10.1126/sciimmunol.adf8161, PMID: 37566678

[18] Cao Z Wang J Liu X Liu Y Li F Liu M. Helminth alleviates covid-19-related cytokine storm in an Il-9-dependent way. mBio. (2024) 15:e0090524. doi: 10.1128/mbio.00905-24, PMID: 38727220 PMC11237724

[19] Scendoni R Bury E Lima Arrais Ribeiro I Cingolani M Cameriere R De Benedictis A. Leading pathogens involved in co-infection and super-infection with covid-19: forensic medicine considerations after a systematic review and meta-analysis. Pathogens. (2023) 12:646. doi: 10.3390/pathogens12050646, PMID: 37242315 PMC10222087

[20] Du Y Wang C Zhang Y. Viral coinfections. Viruses. (2022) 14:2645. doi: 10.3390/v14122645, PMID: 36560647 PMC9784482

[21] Dong L Xing L. Editorial: the biological mechanism and health effect of co-infection with multiple pathogens. Front Cell Infect Microbiol. (2024) 14:1370067. doi: 10.3389/fcimb.2024.1370067, PMID: 38357443 PMC10864655

[22] Bolger AM Lohse M Usadel B. Trimmomatic: A flexible trimmer for illumina sequence data. Bioinformatics. (2014) 30:2114–20. doi: 10.1093/bioinformatics/btu170, PMID: 24695404 PMC4103590

[23] Howe KL Achuthan P Allen J Allen J Alvarez-Jarreta J Amode MR. Ensembl 2021. Nucleic Acids Res. (2021) 49:D884–D91. doi: 10.1093/nar/gkaa942, PMID: 33137190 PMC7778975

[24] Dobin A Davis CA Schlesinger F Drenkow J Zaleski C Jha S. Star: ultrafast universal rna-seq aligner. Bioinformatics. (2013) 29:15–21. doi: 10.1093/bioinformatics/bts635, PMID: 23104886 PMC3530905

[25] Anders S Huber W. Differential expression analysis for sequence count data. Genome Biol. (2010) 11:R106. doi: 10.1186/gb-2010-11-10-r106, PMID: 20979621 PMC3218662

[26] Rosa BA Martin J Mitreva M. R scripts for user-friendly Deseq2 rna-seq differential expression analysis. (2024). doi: 10.21203/rs.3.pex-2532/v1

[27] Katz K Shutov O Lapoint R Kimelman M Brister JR O’Sullivan C. The sequence read archive: A decade more of explosive growth. Nucleic Acids Res. (2022) 50:D387–D90. doi: 10.1093/nar/gkab1053, PMID: 34850094 PMC8728234

[28] Kanehisa M Furumichi M Tanabe M Sato Y Morishima K. Kegg: new perspectives on genomes, pathways, diseases and drugs. Nucleic Acids Res. (2017) 45:D353–D61. doi: 10.1093/nar/gkw1092, PMID: 27899662 PMC5210567

[29] Mitchell AL Attwood TK Babbitt PC Blum M Bork P Bridge A. Interpro in 2019: improving coverage, classification and access to protein sequence annotations. Nucleic Acids Res. (2018) 47:D351–D60. doi: 10.1093/nar/gky1100, PMID: 30398656 PMC6323941

[30] Ge SX Jung D Yao R. Shinygo: A graphical gene-set enrichment tool for animals and plants. Bioinformatics. (2020) 36:2628–9. doi: 10.1093/bioinformatics/btz931, PMID: 31882993 PMC7178415

[31] Gene Ontology C. The gene ontology resource: enriching a gold mine. Nucleic Acids Res. (2021) 49:D325–D34. doi: 10.1093/nar/gkaa1113, PMID: 33290552 PMC7779012

[32] Altschul SF Gish W Miller W Myers EW Lipman DJ. Basic local alignment search tool. J Mol Biol. (1990) 215:403–10. doi: 10.1016/S0022-2836(05)80360-2, PMID: 2231712

[33] Newman AM Steen CB Liu CL Gentles AJ Chaudhuri AA Scherer F. Determining cell type abundance and expression from bulk tissues with digital cytometry. Nat Biotechnol. (2019) 37:773–82. doi: 10.1038/s41587-019-0114-2, PMID: 31061481 PMC6610714

[34] Newman AM Liu CL Green MR Gentles AJ Feng W Xu Y. Robust enumeration of cell subsets from tissue expression profiles. Nat Methods. (2015) 12:453–7. doi: 10.1038/nmeth.3337, PMID: 25822800 PMC4739640

[35] Imai M Iwatsuki-Horimoto K Hatta M Loeber S Halfmann PJ Nakajima N. Syrian hamsters as a small animal model for Sars-Cov-2 infection and countermeasure development. Proc Natl Acad Sci U.S.A. (2020) 117:16587–95. doi: 10.1073/pnas.2009799117, PMID: 32571934 PMC7368255

[36] Shou S Liu M Yang Y Kang N Song Y Tan D. Animal models for covid-19: hamsters, mouse, ferret, mink, tree shrew, and non-human primates. Front Microbiol. (2021) 12:626553. doi: 10.3389/fmicb.2021.626553, PMID: 34531831 PMC8438334

[37] McMahan K Giffin V Tostanoski LH Chung B Siamatu M Suthar MS. Reduced pathogenicity of the Sars-Cov-2 omicron variant in hamsters. Med. (2022) 3:262–8 e4. doi: 10.1016/j.medj.2022.03.004, PMID: 35313451 PMC8926874

[38] Francis ME Goncin U Kroeker A Swan C Ralph R Lu Y. Sars-Cov-2 infection in the Syrian hamster model causes inflammation as well as type I interferon dysregulation in both respiratory and non-respiratory tissues including the heart and kidney. PloS Pathog. (2021) 17:e1009705. doi: 10.1371/journal.ppat.1009705, PMID: 34265022 PMC8282065

[39] Chan JF Zhang AJ Yuan S Poon VK Chan CC Lee AC. Simulation of the clinical and pathological manifestations of coronavirus disease 2019 (Covid-19) in a golden Syrian hamster model: implications for disease pathogenesis and transmissibility. Clin Infect Dis. (2020) 71:2428–46. doi: 10.1093/cid/ciaa325, PMID: 32215622 PMC7184405

[40] Bagato O Balkema-Buschmann A Todt D Weber S Gomer A Qu B. Spatiotemporal analysis of Sars-Cov-2 infection reveals an expansive wave of monocyte-derived macrophages associated with vascular damage and virus clearance in hamster lungs. Microbiol Spectr. (2024) 12:e0246923. doi: 10.1128/spectrum.02469-23, PMID: 38009950 PMC10782978

[41] Zhang J Xu E Ren C Yan W Zhang M Chen M. Mice deficient in Rbm38, a target of the P53 family, are susceptible to accelerated aging and spontaneous tumors. Proc Natl Acad Sci U.S.A. (2014) 111:18637–42. doi: 10.1073/pnas.1415607112, PMID: 25512531 PMC4284600

[42] Yao Y Sun H Chen Y Tian L Huang D Liu C. Rbm24 inhibits the translation of Sars-Cov-2 polyproteins by targeting the 5′-untranslated region. Antiviral Res. (2023) 209:105478. doi: 10.1016/j.antiviral.2022.105478, PMID: 36464077 PMC9712144

[43] Juliana CA Yang J Rozo AV Good A Groff DN Wang SZ. Atf5 regulates beta-cell survival during stress. Proc Natl Acad Sci U.S.A. (2017) 114:1341–6. doi: 10.1073/pnas.1620705114, PMID: 28115692 PMC5307469

[44] Sears TK Angelastro JM. The transcription factor atf5: role in cellular differentiation, stress responses, and cancer. Oncotarget. (2017) 8:84595–609. doi: 10.18632/oncotarget.21102, PMID: 29137451 PMC5663623

[45] Gasser R Cloutier M Prevost J Fink C Ducas E Ding S. Major role of Igm in the neutralizing activity of convalescent plasma against Sars-Cov-2. Cell Rep. (2021) 34:108790. doi: 10.1016/j.celrep.2021.108790, PMID: 33596407 PMC7874916

[46] Sadowska-Bartosz I Bartosz G. Peroxiredoxin 2: an important element of the antioxidant defense of the erythrocyte. Antioxid (Basel). (2023) 12:1012. doi: 10.3390/antiox12051012, PMID: 37237878 PMC10215765

[47] Castro CD Flajnik MF. Putting J chain back on the map: how might its expression define plasma cell development? J Immunol. (2014) 193:3248–55. doi: 10.4049/jimmunol.1400531, PMID: 25240020 PMC4198949

[48] Kawasaki K Ohta Y Castro CD Flajnik MF. The immunoglobulin J chain is an evolutionarily co-opted chemokine. Proc Natl Acad Sci. (2024) 121:e2318995121. doi: 10.1073/pnas.2318995121, PMID: 38215184 PMC10801876

[49] Esmat K Jamil B Kheder RK Kombe Kombe AJ Zeng W Ma H. Immunoglobulin a response to Sars-Cov-2 infection and immunity. Heliyon. (2024) 10:e24031. doi: 10.1016/j.heliyon.2024.e24031, PMID: 38230244 PMC10789627

[50] Branco ACCC Rogers LM Aronoff DM. Folate receptor beta signaling in the regulation of macrophage antimicrobial immune response: A scoping review. Biomed Hub. (2024) 9:31–7. doi: 10.1159/000536186, PMID: 38406385 PMC10890800

[51] Ma J Zhen X Huang X Jiang X. Folic acid supplementation repressed hypoxia-induced inflammatory response via ros and Jak2/Stat3 pathway in human promyelomonocytic cells. Nutr Res. (2018) 53:40–50. doi: 10.1016/j.nutres.2018.03.007, PMID: 29685624

[52] Samblas M Martínez JA Milagro F. Folic acid improves the inflammatory response in lps-activated thp-1 macrophages. Mediators Inflammation. (2018) 2018:1312626. doi: 10.1155/2018/1312626, PMID: 30116142 PMC6079441

[53] Moon SH Liu X Cedars AM Yang K Kiebish MA Joseph SM. Heart failure-induced activation of phospholipase Ipla(2)Gamma generates hydroxyeicosatetraenoic acids opening the mitochondrial permeability transition pore. J Biol Chem. (2018) 293:115–29. doi: 10.1074/jbc.RA117.000405, PMID: 29158256 PMC5766913

[54] Ghosh M Loper R Ghomashchi F Tucker DE Bonventre JV Gelb MH. Function, activity, and membrane targeting of cytosolic phospholipase a(2)Zeta in mouse lung fibroblasts. J Biol Chem. (2007) 282:11676–86. doi: 10.1074/jbc.M608458200, PMID: 17293613 PMC2678067

[55] Chalkidi N Paraskeva C Koliaraki V. Fibroblasts in intestinal homeostasis, damage, and repair. Front Immunol. (2022) 13:924866. doi: 10.3389/fimmu.2022.924866, PMID: 36032088 PMC9399414

[56] Kikut J Mokrzycka M Drozd A Grzybowska-Chlebowczyk U Zietek M Szczuko M. Involvement of proinflammatory arachidonic acid (Ara) derivatives in Crohn’s disease (Cd) and ulcerative colitis (Uc). J Clin Med. (2022) 11:1861. doi: 10.3390/jcm11071861, PMID: 35407469 PMC8999554

[57] Entwistle LJ Pelly VS Coomes SM Kannan Y Perez-Lloret J Czieso S. Epithelial-cell-derived phospholipase a(2) group 1b is an endogenous anthelmintic. Cell Host Microbe. (2017) 22:484–93.e5. doi: 10.1016/j.chom.2017.09.006, PMID: 29024642 PMC5644720

[58] Nguyen HC Bu S Nikfarjam S Rasheed B Michels DCR Singh A. Loss of fatty acid binding protein 3 ameliorates lipopolysaccharide-induced inflammation and endothelial dysfunction. J Biol Chem. (2023) 299:102921. doi: 10.1016/j.jbc.2023.102921, PMID: 36681124 PMC9988587

[59] Assante G Tourna A Carpani R Ferrari F Prati D Peyvandi F. Reduced circulating Fabp2 in patients with moderate to severe Covid-19 may indicate enterocyte functional change rather than cell death. Sci Rep. (2022) 12:18792. doi: 10.1038/s41598-022-23282-x, PMID: 36335131 PMC9637119

[60] Court MH Hazarika S Krishnaswamy S Finel M Williams JA. Novel polymorphic human udp-glucuronosyltransferase 2a3: cloning, functional characterization of enzyme variants, comparative tissue expression, and gene induction. Mol Pharmacol. (2008) 74:744–54. doi: 10.1124/mol.108.045500, PMID: 18523138 PMC2574548

[61] Li J Chen Y Li R Zhang X Chen T Mei F. Gut microbial metabolite hyodeoxycholic acid targets the Tlr4/Md2 complex to attenuate inflammation and protect against sepsis. Mol Ther. (2023) 31:1017–32. doi: 10.1016/j.ymthe.2023.01.018, PMID: 36698311 PMC10124078

[62] Markovich D. Na+-sulfate cotransporter Slc13a1. Pflugers Arch. (2014) 466:131–7. doi: 10.1007/s00424-013-1388-8, PMID: 24193406

[63] Dawson PA Huxley S Gardiner B Tran T McAuley JL Grimmond S. Reduced mucin sulfonation and impaired intestinal barrier function in the hyposulfataemic nas1 null mouse. Gut. (2009) 58:910–9. doi: 10.1136/gut.2007.147595, PMID: 19201772

[64] Szafran BN Borazjani A Scheaffer HL Crow JA McBride AM Adekanye O. Carboxylesterase 1d inactivation augments lung inflammation in mice. ACS Pharmacol Transl Sci. (2022) 5:919–31. doi: 10.1021/acsptsci.2c00098, PMID: 36268116 PMC9578131

[65] Lian J Nelson R Lehner R. Carboxylesterases in lipid metabolism: from mouse to human. Protein Cell. (2018) 9:178–95. doi: 10.1007/s13238-017-0437-z, PMID: 28677105 PMC5818367

[66] Yan D Ye S He Y Wang S Xiao Y Xiang X. Fatty acids and lipid mediators in inflammatory bowel disease: from mechanism to treatment. Front Immunol. (2023) 14:1286667. doi: 10.3389/fimmu.2023.1286667, PMID: 37868958 PMC10585177

[67] Lejeune J Brachet G Watier H. Evolutionary story of the low/medium-affinity Igg fc receptor gene cluster. Front Immunol. (2019) 10:1297. doi: 10.3389/fimmu.2019.01297, PMID: 31244843 PMC6563257

[68] Junqueira C Crespo A Ranjbar S de Lacerda LB Lewandrowski M Ingber J. Fcgammar-mediated Sars-Cov-2 infection of monocytes activates inflammation. Nature. (2022) 606:576–84. doi: 10.1038/s41586-022-04702-4, PMID: 35385861 PMC10071495

[69] Fujiwara RT Cancado GG Freitas PA Santiago HC Massara CL Dos Santos Carvalho O. Necator americanus infection: A possible cause of altered dendritic cell differentiation and eosinophil profile in chronically infected individuals. PloS Negl Trop Dis. (2009) 3:e399. doi: 10.1371/journal.pntd.0000399, PMID: 19308259 PMC2654967

[70] Missailidis D Ebrahimie E Dehcheshmeh MM Allan C Sanislav O Fisher P. A blood-based mrna signature distinguishes people with long covid from recovered individuals. Front Immunol. (2024) 15:1450853. doi: 10.3389/fimmu.2024.1450853, PMID: 39691709 PMC11649547

[71] Bollavaram K Leeman TH Lee MW Kulkarni A Upshaw SG Yang J. Multiple sites on Sars-Cov-2 spike protein are susceptible to proteolysis by cathepsins B, K, L, S and V. Protein Sci. (2021) 30:1131–43. doi: 10.1002/pro.4073, PMID: 33786919 PMC8138523

[72] Srivastava M Steinwede K Kiviranta R Morko J Hoymann HG Langer F. Overexpression of cathepsin K in mice decreases collagen deposition and lung resistance in response to bleomycin-induced pulmonary fibrosis. Respir Res. (2008) 9:54. doi: 10.1186/1465-9921-9-54, PMID: 18638383 PMC2490691

[73] Arampatzidou M Schütte A Hansson GC Saftig P Brix K. Effects of cathepsin K deficiency on intercellular junction proteins, luminal mucus layers, and extracellular matrix constituents in the mouse colon. Biol Chem. (2012) 393:1391–403. doi: 10.1515/hsz-2012-0204, PMID: 23152408 PMC4631841

[74] De Lorenzo R Sciorati C Lore NI Capobianco A Tresoldi C Cirillo DM. Chitinase-3-like protein-1 at hospital admission predicts covid-19 outcome: A prospective cohort study. Sci Rep. (2022) 12:7606. doi: 10.1038/s41598-022-11532-x, PMID: 35534648 PMC9084263

[75] Kamle S Ma B Lee CM Schor G Zhou Y Lee CG. Host chitinase 3-like-1 is a universal therapeutic target for Sars-Cov-2 viral variants in Covid-19. Elife. (2022) 11:e78273. doi: 10.7554/eLife.78273, PMID: 35735790 PMC9273216

[76] Curtiss ML Rosenberg AF Scharer CD Mousseau B Benavides NAB Bradley JE. Chitinase-3-like 1 regulates T(H)2 cells, T(Fh) cells and ige responses to helminth infection. Front Immunol. (2023) 14:1158493. doi: 10.3389/fimmu.2023.1158493, PMID: 37575256 PMC10415220

[77] Mizoguchi E. Chitinase 3-like-1 exacerbates intestinal inflammation by enhancing bacterial adhesion and invasion in colonic epithelial cells. Gastroenterology. (2006) 130:398–411. doi: 10.1053/j.gastro.2005.12.007, PMID: 16472595

[78] Loukas A Hotez PJ Diemert D Yazdanbakhsh M McCarthy JS Correa-Oliveira R. Hookworm infection. Nat Rev Dis Primers. (2016) 2:16088. doi: 10.1038/nrdp.2016.88, PMID: 27929101

[79] Mantovani S Oliviero B Varchetta S Renieri A Mondelli MU. Tlrs: innate immune sentries against Sars-Cov-2 infection. Int J Mol Sci. (2023) 24:8065. doi: 10.3390/ijms24098065, PMID: 37175768 PMC10178469

[80] Ryter SW. Heme oxygenase-1: an anti-inflammatory effector in cardiovascular, lung, and related metabolic disorders. Antioxid (Basel). (2022) 11:555. doi: 10.3390/antiox11030555, PMID: 35326205 PMC8944973

[81] Hara Y Tsukiji J Yabe A Onishi Y Hirose H Yamamoto M. Heme oxygenase-1 as an important predictor of the severity of Covid-19. PloS One. (2022) 17:e0273500. doi: 10.1371/journal.pone.0273500, PMID: 36001619 PMC9401165

[82] Batra N De Souza C Batra J Raetz AG Yu AM. The hmox1 pathway as a promising target for the treatment and prevention of Sars-Cov-2 of 2019 (Covid-19). Int J Mol Sci. (2020) 21:6412. doi: 10.3390/ijms21176412, PMID: 32899231 PMC7503392

[83] Lujan RA Pei L Shannon JP Dábilla N Dolan PT Hickman HD. Widespread and dynamic expression of granzyme C by skin-resident antiviral T cells. Front Immunol. (2023) 14:1236595. doi: 10.3389/fimmu.2023.1236595, PMID: 37809077 PMC10552530

[84] de Jong LC Crnko S Ten Broeke T Bovenschen N. Noncytotoxic functions of killer cell granzymes in viral infections. PloS Pathog. (2021) 17:e1009818. doi: 10.1371/journal.ppat.1009818, PMID: 34529743 PMC8445437

[85] Nagozir S Shakouri Khomartash M Parsania M Vahidi M Ghorbani M. Association between genetic variants in the Cd209 gene and susceptibility to covid-19 in Iranian population. Hum Gene. (2023) 38:201215. doi: 10.1016/j.humgen.2023.201215

[86] Ficial M Antonaglia C Chilosi M Santagiuliana M Tahseen AO Confalonieri D. Keratin-14 expression in pneumocytes as a marker of lung regeneration/repair during diffuse alveolar damage. Am J Respir Crit Care Med. (2014) 189:1142–5. doi: 10.1164/rccm.201312-2134LE, PMID: 24787069

[87] Chang CY Huang YC Chiang HH Wu YY Wu KL Chang YY. Ladinin 1 shortens survival via promoting proliferation and enhancing invasiveness in lung adenocarcinoma. Int J Mol Sci. (2022) 24:431. doi: 10.3390/ijms24010431, PMID: 36613882 PMC9820746

[88] Synolaki E Papadopoulos V Divolis G Tsahouridou O Gavriilidis E Loli G. The activin/follistatin axis is severely deregulated in Covid-19 and independently associated with in-hospital mortality. J Infect Dis. (2021) 223:1544–54. doi: 10.1093/infdis/jiab108, PMID: 33625513 PMC7928794

[89] Group P-CC. Clinical characteristics with inflammation profiling of long covid and association with 1-year recovery following hospitalisation in the UK: A prospective observational study. Lancet Respir Med. (2022) 10:761–75. doi: 10.1016/S2213-2600(22)00127-8, PMID: 35472304 PMC9034855

[90] Warren DS Morrell JC Moser HW Valle D Gould SJ. Identification of Pex10, the gene defective in complementation group 7 of the peroxisome-biogenesis disorders. Am J Hum Genet. (1998) 63:347–59. doi: 10.1086/301963, PMID: 9683594 PMC1377304

[91] Kronstein-Wiedemann R Stadtmuller M Traikov S Georgi M Teichert M Yosef H. Sars-Cov-2 infects red blood cell progenitors and dysregulates hemoglobin and iron metabolism. Stem Cell Rev Rep. (2022) 18:1809–21. doi: 10.1007/s12015-021-10322-8, PMID: 35181867 PMC8856880

[92] Byrne A Manalo G Clarke NE Vaz Nery S. Impact of hookworm infection and preventive chemotherapy on haemoglobin in non-pregnant populations. Trop Med Int Health. (2021) 26:1568–92. doi: 10.1111/tmi.13681, PMID: 34587315

[93] Tong M Jiang Y Xia D Xiong Y Zheng Q Chen F. Elevated expression of serum endothelial cell adhesion molecules in Covid-19 patients. J Infect Dis. (2020) 222:894–8. doi: 10.1093/infdis/jiaa349, PMID: 32582936 PMC7337874

[94] Wu S Xu Y Zhang J Ran X Jia X Wang J. Longitudinal serum proteome characterization of covid-19 patients with different severities revealed potential therapeutic strategies. Front Immunol. (2022) 13:893943. doi: 10.3389/fimmu.2022.893943, PMID: 35958562 PMC9361788

[95] Zhu R Chen Y-T Wang B-W You Y-Y Wang X-H Xie H-T. Tap1, a potential immune-related prognosis biomarker with functional significance in uveal melanoma. BMC Cancer. (2023) 23:146. doi: 10.1186/s12885-023-10527-9, PMID: 36774490 PMC9921415

[96] Ning S Pagano JS Barber GN. Irf7: activation, regulation, modification and function. Genes Immun. (2011) 12:399–414. doi: 10.1038/gene.2011.21, PMID: 21490621 PMC4437765

[97] Zhang Y Wang J Yu C Xia K Yang B Zhang Y. Advances in single-cell sequencing and its application to musculoskeletal system research. Cell Prolif. (2022) 55:e13161. doi: 10.1111/cpr.13161, PMID: 34888976 PMC8780907

[98] Betancor G. You shall not pass: Mx2 proteins are versatile viral inhibitors. Vaccines (Basel). (2023) 11:930. doi: 10.3390/vaccines11050930, PMID: 37243034 PMC10224399

[99] Bizzotto J Sanchis P Abbate M Lage-Vickers S Lavignolle R Toro A. Sars-Cov-2 infection boosts mx1 antiviral effector in covid-19 patients. iScience. (2020) 23:101585. doi: 10.1016/j.isci.2020.101585, PMID: 32989429 PMC7510433

[100] Harioudh MK Perez J Chong Z Nair S So L McCormick KD. Oligoadenylate synthetase 1 displays dual antiviral mechanisms in driving translational shutdown and protecting interferon production. Immunity. (2024) 57:446–61 e7. doi: 10.1016/j.immuni.2024.02.002, PMID: 38423012 PMC10939734

[101] Jain S Rego S Park S Liu Y Parn S Savsani K. Rnaseq profiling of covid19-infected patients identified an Eif2ak2 inhibitor as a potent Sars-Cov-2 antiviral. Clin Transl Med. (2022) 12:e1098. doi: 10.1002/ctm2.1098, PMID: 36321336 PMC9627224

[102] Tolomeo M Cavalli A Cascio A. Stat1 and its crucial role in the control of viral infections. Int J Mol Sci. (2022) 23:4095. doi: 10.3390/ijms23084095, PMID: 35456913 PMC9028532

[103] Huang M Mark A Pham J Vera K Saravia-Butler AM Beheshti A. Rna editing regulates host immune response and T cell homeostasis in Sars-Cov-2 infection. PloS One. (2024) 19:e0307450. doi: 10.1371/journal.pone.0307450, PMID: 39178184 PMC11343423

[104] Boudewijns R Thibaut HJ Kaptein SJF Li R Vergote V Seldeslachts L. Stat2 signaling restricts viral dissemination but drives severe pneumonia in Sars-Cov-2 infected hamsters. Nat Commun. (2020) 11:5838. doi: 10.1038/s41467-020-19684-y, PMID: 33203860 PMC7672082

[105] Zhao YC Wang TJ Cui J She LZ Zhang RF Zhang CH. The role of Slc39a4 in the prognosis, immune microenvironment, and contribution to Malignant behavior *in vivo* and *in vitro* of cervical cancer. Transl Oncol. (2024) 40:101839. doi: 10.1016/j.tranon.2023.101839, PMID: 38029507 PMC10698533

[106] Smith JL Brooker S. Impact of hookworm infection and deworming on anaemia in non-pregnant populations: A systematic review. Trop Med Int Health. (2010) 15:776–95. doi: 10.1111/j.1365-3156.2010.02542.x, PMID: 20500563 PMC2916221

[107] Dunaway LS Loeb SA Petrillo S Tolosano E Isakson BE. Heme metabolism in nonerythroid cells. J Biol Chem. (2024) 300:107132. doi: 10.1016/j.jbc.2024.107132, PMID: 38432636 PMC10988061

[108] Kaneko K Furuyama K Fujiwara T Kobayashi R Ishida H Harigae H. Identification of a novel erythroid-specific enhancer for the alas2 gene and its loss-of-function mutation which is associated with congenital sideroblastic anemia. Haematologica. (2014) 99:252–61. doi: 10.3324/haematol.2013.085449, PMID: 23935018 PMC3912954

[109] Kant TA Newe M Winter L Hoffmann M Kammerer S Klapproth E. Genetic deletion of polo-like kinase 2 induces a pro-fibrotic pulmonary phenotype. Cells. (2021) 10:617. doi: 10.3390/cells10030617, PMID: 33799608 PMC8001503

[110] Di Vito C Calcaterra F Coianiz N Terzoli S Voza A Mikulak J. Natural killer cells in Sars-Cov-2 infection: pathophysiology and therapeutic implications. Front Immunol. (2022) 13:888248. doi: 10.3389/fimmu.2022.888248, PMID: 35844604 PMC9279859

[111] Park YJ Acosta D Rubel Hoq M Khurana S Golding H Zaitseva M. Pyrogenic and inflammatory mediators are produced by polarized M1 and M2 macrophages activated with D-dimer and Sars-Cov-2 spike immune complexes. Cytokine. (2024) 173:156447. doi: 10.1016/j.cyto.2023.156447, PMID: 38041875

[112] Meneses-Preza YG Soria-Castro R Alfaro-Doblado AR Hernandez-Solis A Alvarez-Maldonado P Gomez-Martin D. Mast cell activation signature as a potential biomarker in Covid-19. Immunol Lett. (2025) 275:107026. doi: 10.1016/j.imlet.2025.107026, PMID: 40250770

[113] Nair MG Herbert DR. Immune polarization by hookworms: taking cues from T helper type 2, type 2 innate lymphoid cells and alternatively activated macrophages. Immunology. (2016) 148:115–24. doi: 10.1111/imm.12601, PMID: 26928141 PMC4863575

[114] Periago MV Bethony JM. Hookworm virulence factors: making the most of the host. Microbes Infect. (2012) 14:1451–64. doi: 10.1016/j.micinf.2012.09.002, PMID: 23006854

