# Supplementary material for: Hookworm infection modulates lung and intestinal transcriptomic responses to SARS-CoV-2 in Syrian hamsters
Source: Front Immunol. 2025 Nov 5;16:1701728. doi: 10.3389/fimmu.2025.1701728 (PMC12627036; doi:10.3389/fimmu.2025.1701728)
Supplement: Supplementary Figure 1 — Counts of significantly enriched Gene Ontology terms + KEGG pathway counts among significantly differentially expressed genes in each infected cohort relative to the uninfected controls, (A) in the lungs and (B) in the intestine. For all enrichment testing, FDR-adjusted P values ≤ 0.05 were used to identify significant enrichment. [file DataSheet1.pdf]

Supplementary Figures

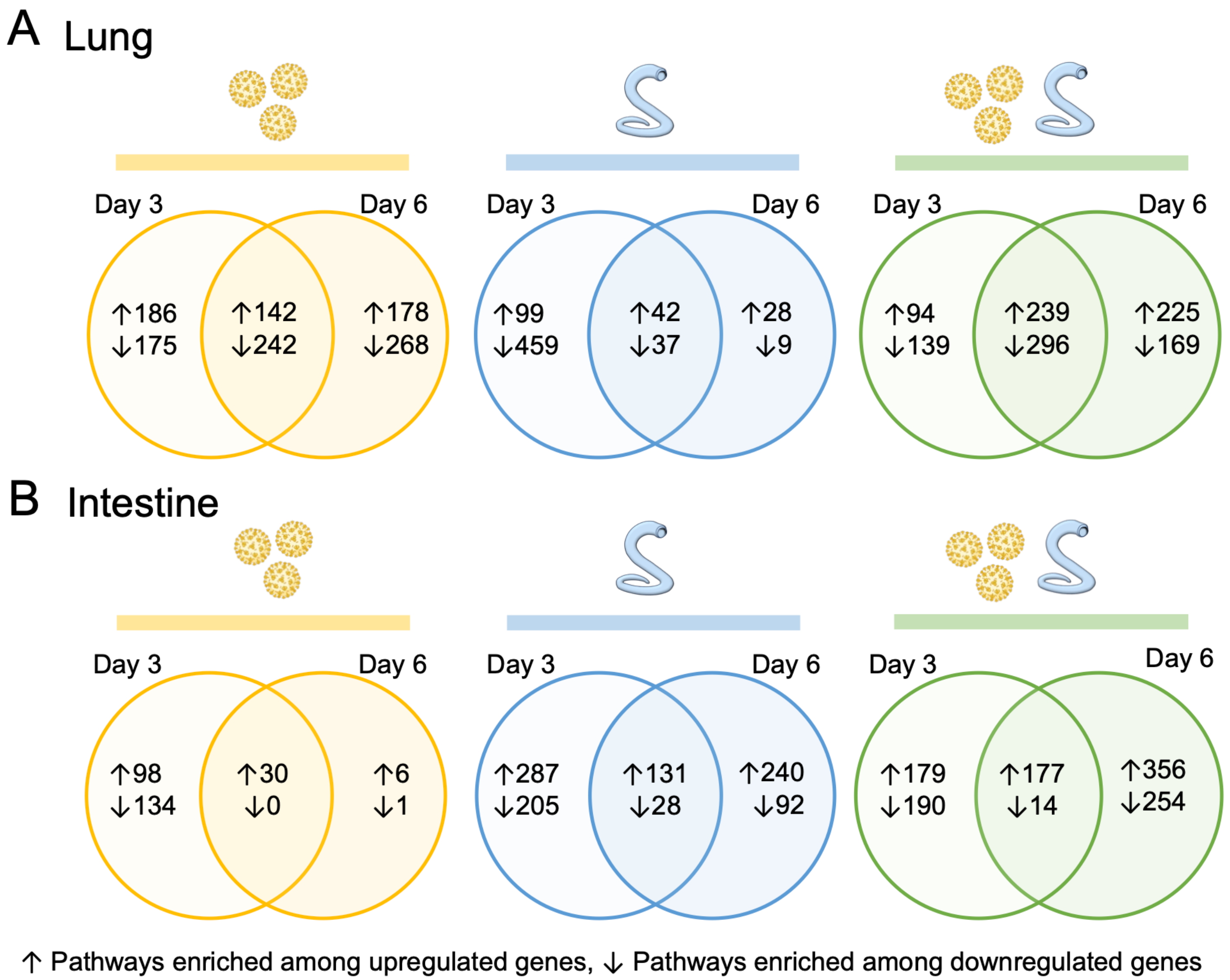

Supplementary Figure S1: Counts of significantly enriched Gene Ontology terms + KEGG pathway counts among significantly differentially expressed genes in each infected cohort relative to the uninfected controls, (**A**) in the lungs and (**B**) in the intestine. For all enrichment testing, FDR-adjusted *P* values ≤ 0.05 were used to identify significant enrichment.

Cell type			Naive Day 6	CoV Day 3	CoV Day 6	HW Day 3	HW Day 6	HW+CoV Day 3	HW+CoV Day 6
			Proportion of cells in lung (average %; inferred by CIBERSORT)						
Lymphoid cells	B cells	Naive	5.1	0.5	0.2	3.1	0.0	0.0	0.0
		Memory	4.7	0.4	1.1	4.6	10.1	2.6	2.6
		Plasma	19.3	10.6	10.5	22.4	26.3	13.4	13.6
	T cells	CD8	0.1	0.0	**2.8***	0.0	0.0	0.1	2.0
		CD4 naive	2.9	0.0	0.0	0.9	3.8	0.0	0.0
		CD4 memory resting	4.0	11.0	5.4	6.3	0.6	9.0	4.8
		CD4 memory activated	0.0	0.0	0.0	0.0	0.0	0.0	0.0
		Follicular helper	2.4	4.3	4.4	2.0	3.3	3.5	7.6
		Regulatory (Tregs)	4.8	0.7	3.1	3.1	2.3	1.1	1.6
		Gamma delta	0.0	**4.6***	2.3	1.2	0.2	**5.5***	3.3
	NK cells	Resting	0.0	0.0	0.0	0.0	0.0	0.0	0.0
		Activated	12.6	**21.1***	**23.2***	**8.3***	9.3	**21.1***	**24.6***
Myeloid / innate immune cells	Monocytes		26.1	25.9	18.7	32.4	29.4	28.6	13.6
	Macro-phages	M0	5.7	**0.0****	**0.0****	7.5	4.5	**0.3***	**0.0****
		M1	1.8	**7.1*****	**6.6****	1.2	1.5	**6.3***	7.6
		M2	0.0	**8.6***	**18.9*****	0.7	0.0	4.5	**15.4***
	Dendritic cells	Resting	3.0	**0.1***	0.3	**0.1***	1.5	0.1	0.6
		Activated	0.0	0.0	0.0	0.0	0.0	0.0	0.0
Granu-locytes and related innate cells	Mast cells	Resting	7.3	**0.3*****	**1.0****	5.6	4.7	**0.0*****	**0.0*****
		Activated	0.0	4.0	1.5	0.0	0.0	3.0	2.4
	Eosinophils		0.0	0.0	0.0	0.0	0.0	0.0	0.0
	Neutrophils		0.0	0.9	0.0	0.4	2.3	0.8	0.3

Significantly higher | Significantly Lower

Supplementary Figure S2: Average estimated relative proportions of immune cell types, estimated by CIBERSORTx. Significance values represent two-tailed T-tests (unequal variance) compared to the Naive cohort, FDR-corrected for multiple test correction across all tests (* $P \leq 0.05$, ** $P \leq 0.01$, *** $P \leq 0.001$). Relative proportions per sample and exact P values are provided in **Supplementary Table 6**. Blue shading = significantly higher, red shading = significantly lower, green shading reflects the relative abundance of each cell type in the naive sample.
